# Adaptive Introgression as an Evolutionary Force: A Meta‐Analysis of Knowledge Trends

**DOI:** 10.1111/eva.70103

**Published:** 2025-06-20

**Authors:** Pedro Horta, Helena Raposeira, Javier Juste, Orly Razgour, Hugo Rebelo

**Affiliations:** ^1^ CIBIO/InBio Centro de Investigação em Biodiversidade e Recursos Genéticos da Universidade do Porto Vairão Portugal; ^2^ Palombar – Conservação da Natureza e do Património Rural Vimioso Portugal; ^3^ OII – Observatório Inovação Investigação Seia Portugal; ^4^ BIOPOLIS Program in Genomics, Biodiversity and Land Planning, CIBIO Campus de Vairão Vairão Portugal; ^5^ Departamento de Ciências da Vida, TERRA Laboratório Associado, Centro de Ecologia Funcional Universidade de Coimbra Coimbra Portugal; ^6^ Departamento de Biologia Faculdade de Ciências, Universidade do Porto Porto Portugal; ^7^ Departamento de Ecología Evolutiva Estación Biológica de Doñana (CSIC) Sevilla Spain; ^8^ CIBER de Epidemiología y Salud Pública, CIBERESP Madrid Spain; ^9^ Biosciences, University of Exeter Exeter UK; ^10^ Faculdade de Ciências, Universidade de Lisboa Lisboa Portugal

**Keywords:** adaptive introgression, biological organization, evolution, meta‐analysis, structural complexity, systematics review, trends in knowledge

## Abstract

There is growing evidence for the role of introgressive hybridization in promoting species adaptation (i.e., adaptive introgression) owing to increasing genomic studies on a diversity of taxa over the past decades. However, introgressive hybridization was, and still is, regarded as a homogenizing process hindering the evolutionary process of adaptation to selection pressures. Despite methodological advances, key gaps remain in understanding how adaptive introgression due to hybridization functions across taxonomic groups and biological levels. This study has three objectives: (1) to explore historical trends in the understanding of adaptive introgression, particularly its genomic and functional dimensions; (2) to investigate structural organismal characteristics influencing patterns of adaptive introgression; and (3) to evaluate how adaptive introgression interacts with counteracting evolutionary mechanisms. We carried out a systematic review of the adaptive introgression literature and a multidimensional meta‐analysis. The current knowledge trends have been shaped by the genomic revolution. Since 2012, genomic studies have contributed to establishing a clearer understanding of adaptive introgression. The amount and variety of published studies increased from bacteria to mammals across a complexity gradient, focusing on the genomic level and progressively having consequences at a greater number of levels of biological organization (from physiological and demographic to behavioral/ecological). Testing for tendencies, our study also revealed evolutionary mechanisms linked to adaptive introgression co‐occurring with divergence forces, demonstrating that these processes are not mutually exclusive, even when they act in opposite directions, i.e., convergence and divergence, such as autosomal introgression (versus islands of differentiation in sex‐linked chromosomes), balancing selection (versus genetic drift), or sexual selection (versus assortative mating). This balance is mediated by environmental conditions as they are frequently reported in the studies, regardless of the organisms' structural complexity, shaping the path of the evolutionary process of introgressing species. Studying introgression patterns has important implications for understanding adaptation in rapidly changing environments.

## 1 Introduction

Repeated interbreeding can result in closely related species sharing genetic material (including both nuclear DNA [nDNA] and/or cytoplasmic DNA [cDNA]) through introgression (Arnold 2004; Hedrick 2013; Powell et al. 1999). This process was historically regarded as a homogenizing process (Arnold 1992; Latch et al. 2006), counteracting local selection pressures and decreasing the fitness of diverging populations by introducing alleles outside the local adaptive range (Bolnick and Nosil 2007; Maguilla et al. 2017). Consequently, it was considered a conservation concern due to the risk of acting against divergence and leading to genetic swamping (see Table 1—Glossary), which could impact species' long‐term survival (Maguilla et al. 2017). Following this rationale, some authors considered introgression as a damage to species integrity, a maladaptive process, or an unexpected misfortune of nature (Llopart et al. 2014).

**TABLE 1:** Glossary.

*Adaptive introgression*
The natural transfer of genetic material by interspecific breeding and backcrossing of hybrids with parental species followed by selection on introgressed alleles (de Lafontaine et al. 2015; Song et al. 2011)
*Assortative mating*
A mating behavior by which individuals show higher preference for mating with individuals with similar phenotypes and genotypes than expected under a random mating pattern (Herbers 2010)
*Balancing selection*
A set of selective pressures which actively allow the maintenance of multiple alleles in the population gene pool at greater frequencies than expected based on the effects of genetic drift alone (King et al. 2006)
*Biological organization*
The hierarchical organization of complex biological structures and systems (Mazzocchi 2008)
*Genetic drift*
Changes in the frequency of an existing allele in the genetic pool of a population as a result of the random sampling of organisms (Bolnick et al. 2011)
*Genetic recombination*
Genetic exchange between different taxa which leads to new combinations of traits that differ from those found in the parental species (Martinsen et al. 2001)
*Genetic swamping*
Gene flow from most abundant species toward species with smaller population size that can lead to outbreeding depression due to the replacement of local genotypes (Todesco et al. 2016)
*Genomic introgression*
Distribution of introgressed alleles in across the genome (Gompert and Buerkle 2009)
*Geographic introgression*
Geographic distribution of alleles across a hybrid zone (Payseur et al. 2004)
*Haldane's rule*
Heterozygous sex shows greater hybrids fitness reduction (Haldane 1922)
*Islands of differentiation*
Islands of differentiation are genomic regions exhibiting unusually high levels of differentiation between populations or species (Nachman and Payseur 2012) that can be directly involved in the process of reproductive isolation and divergence or not (Nosil et al. 2012)
*Natural hybridization*
Secondary breeding contact between two populations that have evolved separately over a period of time but under natural conditions (Neri et al. 2017)
*Selective sweep*
Genetic process through which a new allele that increases the fitness of the individual increases in frequency in a population and becomes fixed (frequency of 1) due to natural selection (Bay and Ruegg 2017; Kuhlwilm et al. 2019)
*Sexual selection*
Breeding behavior favoring mating partners with adaptive advantage (higher fitness), independently of their species (Parrett and Knell 2018)
*Transgressive segregation*
Extreme phenotypic traits outside the original phenotypic range of parental species produce by introgression and leading to hybrid speciation (Nichols et al. 2015; Rieseberg et al. 2003)

On the other hand, since the publication of the first theoretical essays on the topic, the role of introgression was the subject of controversy because introgressed alleles can be found in parts of the genome that are under selection as well as neutral regions, and may contribute to adaptations, have no impact, or be eliminated for being deleterious (introgression suppression) (Seixas et al. 2018). Introgression is considered neutral when introgressed alleles have no phenotypic/physiological consequences that could affect the fitness of the receptor lineage/species (Matosiuk et al. 2014). Otherwise, it is considered maladaptive if it has negative adaptive outcomes by reducing the fitness or survival of an evolutionary lineage in its environment (Minder and Widmer 2008). Factors such as changes in population size, mating system, and recombination rate can have different outcomes and influence the accumulation of deleterious genetic variation in natural populations. Deleterious variations—whether recessive or additive—can confound the detection of adaptive introgression (Kim et al. 2018).

However, contrary to the historical assumption that introgression has mainly neutral impacts (Van Valen 1976; Räsänen and Hendry 2008), recent evidence supports that beneficial alleles introgress more easily than neutral ones (Harrison and Larson 2014). Random processes, like genetic drift (Gompert et al. 2013; Wade and Goodnight 2006) can eliminate maladaptive and deleterious alleles in a few generations or keep others with the propensity to persist as neutral and dependent on demography, following the selection process through which maladaptive and deleterious alleles tend to be eliminated. Neutral alleles are only subject to genetic drift, while favorable alleles tend to increase in frequency (Pease et al. 2016). This selective process led to adaptive introgression (see Table 1—Glossary) being increasingly documented as a major adaptive driving force leading to faster adaptation than *de novo* mutations (Pfeifer and Kapan 2019).

An increasing volume of evidence has demonstrated that adaptation to novel conditions can occur by the introgression of beneficial alleles (Jones et al. 2018; Lexer et al. 2004; Nadachowska‐Brzyska et al. 2012). Adaptive introgression enhances adaptive capacity and drives evolutionary leaps, bypassing intermediate evolutionary stages (Acosta and Premoli 2018). Thus, faster adaptation can occur when this standing genetic variation comes from other species that are better adapted to the environmental conditions. This mechanism can increase species survival (Liu et al. 2015; Norris et al. 2015), promoting their range expansion (Aleksic et al. 2018), and even supporting evolutionary rescue (Aeschbacher et al. 2017). Paradoxically, in some circumstances, adaptive introgression can even lead to species divergence (Alexander et al. 2017; Antelope et al. 2017), for example, by promoting hybrid speciation through transgressive segregation (Arnold et al. 2012).

Being rare, beneficial introgressed alleles can be favored, for example, by balancing selection (Nadachowska‐Brzyska et al. 2012), and quickly establish in the receptor species through selective sweeps (Bay and Ruegg 2017; Kuhlwilm et al. 2019). Unlike new mutations, which start with a prevalence of 1/2 N, the introgressed alleles may have a higher “initial” prevalence, depending on hybridization rates (Pfeifer and Kapan 2019). Thus, a high initial prevalence of introgressed alleles can also facilitate their fixation, regardless of the fitness effect. After settling stably, these alleles can act as an important source of novel genetic and phenotypic variation at some point in evolutionary history (Baldassarre et al. 2014; Cahill et al. 2015). Just a single introgressed gene can have the potential to promote variation and consequently have a large ecological impact (Bradshaw and Schemske 2003).

The advent of high‐throughput sequencing approaches resulted in the exponential growth of scientific evidence documenting the occurrence of introgression and, to a lesser extent, its adaptive and evolutionary consequences (Taylor and Larson 2019). From the global set of literature, adaptive introgression stands out as an extremely complex process that acts across divergence processes in all taxonomic groups spanning various levels of structural complexity, including bacteria (Palmer et al. 2010), protists (Nader et al. 2019), fungi (Dunn et al. 2013), plants, from bryophytes (James et al. 2008) to angiosperms (Taylor et al. 2009), and animals, both invertebrates (Norris et al. 2015) and vertebrates (Liu et al. 2015). Environmental pressures, both natural (Matosiuk et al. 2014) and anthropogenic (Pardo‐Diaz et al. 2012), drive adaptive introgression at the genomic level, leading to consequences at different levels of biological organization (see Table 1—Glossary), from physiology (Storchová et al. 2004) to behavior (McDonald et al. 2001) and demography (Ray and Excoffier 2009). Understanding how adaptive introgression may play a vital role as an evolutionary force will require a vast review of the state of knowledge on the adaptive introgression process across the tree of life and in all its biological dimensions (Pfennig 2021). Systematic reviews and meta‐analyses merge and evaluate large datasets, providing insights into trends and knowledge gaps, providing a foundation for future research on adaptation in rapidly changing environments (O'Dea et al. 2021). Standard protocols of systematic reviews allow for reproducibility and prevent against subjective decision‐making during the review process (Page et al. 2021; Shamseer et al. 2015). Meta‐analyses through multidimensional network approaches provide an integrative and holistic framework to merge biological data from standard systematic reviews (Bradley et al. 2005). They can help to extend the understanding of the evolutionary dynamics that drive species' adaptation at different levels of biological organization.

As research on adaptive introgression expands rapidly, a systematic review is needed to synthesize and evaluate this growing body of evidence. This study aims to examine the biological dimensions of adaptive introgression through a multidimensional network meta‐analysis. Characteristics intrinsic to organisms—such as cellular organization (prokaryotic or eukaryotic), the presence of sex‐linked chromosomes, and patterns of sexual selection—likely play a significant role in shaping adaptive traits arising from introgression. Similarly, genomic architecture plays a fundamental role in shaping the likelihood of adaptive introgression. Structural genomic features, especially in terms of genetic incompatibilities and structural variation, such as chromosomal inversions, translocations, and fusions, can act as barriers to gene flow by reducing recombination or promoting reproductive isolation (Hoffmann and Rieseberg 2008; Feder et al. 2012).

Adaptive introgression does not occur in isolation; it is countered and modulated by other evolutionary mechanisms associated with species divergence, including genetic incompatibility and assortative mating. For instance, islands of differentiation—defined as genomic regions exhibiting unusually high levels of differentiation between populations or species (Nachman and Payseur 2012)—can act as barriers to gene flow, either by reinforcing reproductive isolation or by providing selective environments that limit or enhance the spread of introgressed alleles. To explore these dynamics, we defined three main objectives: (1) to explore historical trends in the understanding of adaptive introgression, particularly its cellular and genomic localization, directional transmission, and potential correlation with other evolutionary mechanisms, like genetic drift or assortative mating; (2) to investigate how characteristics of organisms, including their structural and functional organizational unit (whether prokaryotic or eukaryotic), as well as the presence/absence of sex‐linked chromosomes and mating strategies, influence patterns on the traits associated with adaptive introgression; (3) evaluating interactions between adaptive introgression and key evolutionary mechanisms arising from divergence that can simultaneously act as counterforces.

To address these objectives, we tested the following hypotheses: (1) Advances in genomics have significantly improved our understanding of adaptive introgression, leading to more stable and clearer knowledge patterns in recent studies, across diverse taxonomic groups and biological levels; (2) More complex organisms—genetically, physiologically, and behaviorally—are expected to show more intricate and diverse patterns of adaptive introgression across biological levels compared to simpler organisms; (3) Adaptive introgression and divergence forces can coexist and act concurrently within populations, even when these processes drive opposing evolutionary outcomes (e.g., convergence versus divergence). Clarifying these objectives and testing these hypotheses could bring us new synthesized views about how introgression can act as an evolutionary force shaping the paths of evolution and offering crucial applied implications for understanding adaptation in rapidly changing environments.

## 2 Materials and Methods

### 2.1 Literature Search

We carried out a systematic review searching for studies that assessed introgression patterns resulting from adaptation. Following the standard PRISMA review protocols (Moher et al. 2009; O'Dea et al. 2021), we used ISI Web of Knowledge, Scopus, Google, and Google Scholar as the search engines (Falagas et al. 2008). We selected a set of search terms (see Supporting Information for details) related to natural hybridization (see Table 1—Glossary), genomics, introgression, and adaptation, intentionally avoiding terms associated with direct or indirect human intervention (e.g., breeding or genetic manipulations), such as domestication and invasive scenarios, limiting the search to natural genomic introgression (see Table 1—Glossary) with potential adaptive consequences. The keywords were selected from a list of keywords in reference papers and through the consultation of a team of experts in evolutionary biology. To obtain the largest number of publications on the topic, the final set of selected keywords included the most common, unambiguous terms and expressions (see Supporting Information for search details). The searches covered a period from 1900 to November 2019 and were restricted to English.

Our searches produced 820 publications. From this dataset, we removed duplicates and excluded theoretical essays, opinion texts, non–peer‐reviewed papers, off‐topic documents, and non‐ISI journals. Then we checked the reference list of the remaining publications looking for additional works that included the terms under study and that were not counted in the initial search. From this global set of publications, we excluded all reviews and book chapters. Both the title and abstract of the remaining 436 articles were read to assess their relevance for the study, based on: (1) if the adaptive introgression process was the result of natural processes, and (2) if adaptive introgression was identified by the authors as the most plausible explanation behind the observed adaptive processes. The final dataset comprised 358 studies (Figure S1—Supporting Information). All papers were analyzed in full by the first author.

### 2.2 Systematic Review

We extracted bibliographic information from the 358 studies, including the temporal period and spatial location of the adaptive introgression phenomena reported in each study, the taxa undergoing the introgression process, and the main study methods (type of experiment—including introgression potentially identified without molecular data by methods like crossbreeding experiments—type of molecular introgression markers, and whether whole‐genome sequencing was performed). Finally, we recorded the main characteristics of introgression referred to in each study and the associated adaptive outcomes. Detailed information about the variables extracted is available in Table S1 (Supporting Information). It is important to consider that it is not always certain that alleles/traits are adaptively introgressed. Therefore, we based this conclusion on the authors' interpretation as stated in the conclusions of the study, and on described evidence of shared genes and/or traits that have potentially adaptive outcomes.

### 2.3 Meta‐Analysis

#### 2.3.1 Temporal Changes in Publication

Temporal changes in the number of published papers were tested through General Linear Models (GLM) by the Expert Modeler Methods available on IBM SPSS Statistics software (v. 22; SPSS Inc., Chicago, IL). This approach was complemented with a linear regression, assessed through the coefficient of determination, between the number of published papers and time. We defined the tipping point as the temporal moment when the exponential trend lines ceased to show a reduced slope (Maroco 2010).

#### 2.3.2 Influence of Structural Characteristics of Organisms on Adaptive Introgression Knowledge

We quantified complexity levels across taxonomic groups by summing binary classifications for three critical characteristics in their evolutionary history. Specifically, we assigned binary values (0 or 1) to each group based on the following criteria: the structural and functional organizational unit (prokaryotic = 0; eukaryotic = 1), the presence or absence of sex‐linked chromosomes (absent = 0; present = 1), and the predominant mating strategies (random = 0; sexual selection = 1). This method provided a standardized metric for assessing complexity, as detailed in Table S2 (Supporting Information). We then organized these groups according to the quantified degree of genetic and behavioral complexity, which is also likely correlated with physiological and ecological complexity, creating a taxonomic complexity gradient available in Figure 2. Examples of complex taxonomic groups included eukaryotic species with sex‐linked chromosomes and complex behavioral mechanisms of sexual selection, such as mammals and angiosperms. We performed a bipartite network analysis using the open‐source graph viz. platform Gephi 0.10 (https://gephi.org/) of adaptive introgression's reported characteristics in different levels of biological organization (Genomics/cytology—Location of introgression/islands of differentiation in the genome/cell; Physiology—Physiological constraints on adaptive introgression patterns; Demography—The impacts of demography on the direction and amount/patterns of adaptive introgression; Behavior—Behavioral impact on adaptive introgression patterns; and Ecology—Ecological influences on adaptive introgression patterns) and the taxonomic groups across the taxonomic complexity gradient.

The significance of the Taxonomic Group factor (A—Prokaryotes, B—Singled‐cell eukaryotes, C—Eukaryotes with sex‐linked chromosomes and random mate, and D—Eukaryotes with sexual selection) on the percentage of connections across the five levels of biological organization (“Genomics/cytology,” “Physiology,” “Demography,” “Behaviour,” and “Ecology”) was assessed using a MANOVA, after validating normality assumptions with the Kolmogorov–Smirnov test (*p* > 0.05 for all groups). The assumption of homogeneity of variances‐covariances was verified with Levene's test. Statistical analysis was performed using SPSS software. When MANOVA detected significant effects, ANOVA was conducted for each variable, followed by Tukey's HSD post hoc test (Table S3—Supporting Information).

#### 2.3.3 Adaptive Introgression and Divergence Forces—Multilayer Network Approach

We performed a multilayer analysis through an open‐source multilayer network visualization and analysis platform MuxViz (http://muxviz.net/). MuxViz is an approach for representing a large variety of complex systems, such as biological structures, at different levels of biological organization, using empirical genetic, neuronal, and transportation networks (De Domenico et al. 2014). Multilayer graphs of networks can be visualized by creating a relationship between two or more extracted variables, as nodes, incorporating them into different levels (layers) (De Domenico et al. 2014). These relationships occur when two characteristics of a case study (two variables extracted) are simultaneously described in the same paper which, depending on whether they are interdependent or not, can be directed or not directed. Here, the graph layers were designed to represent the different levels of biological organization: (1) Genomics/cytology; (2) Physiology; (3) Behavior; (4) Demography; and (5) Ecology. We used the Large Graph Layout (LGL) algorithm at two dimensions with multiplex node arrangement and the *interception* as the template method, where all nodes can be connected (intra and inter‐layer connection) (De Domenico et al. 2014).

For computing multilayer‐specific network properties, we performed an eigenvector centrality algorithm to analyze eigenvector versatility through the multilayer extension, which measures each node's importance (standardized between 0 and 1) depending on its connections within and across layers (De Domenico et al. 2014). Node size was represented by the algorithm PageRank centrality (standardized between 0 and 1) that rank case studies' characteristics (see Supporting Information) to quantify the importance of each node in the network by correlation with eigenvector metrics (De Domenico et al. 2015).

To standardize the intensity of connections between nodes, we used normalization techniques. For the exploratory analysis, we calculated percentages based on dividing the number of connections identified between two nodes by the total number of distinct types of connections present in the database. This database encompasses a total of 210 different edges, serving as the entire universe for our analysis. Furthermore, we devised a method to assess the strength of connections between nodes by introducing an Edge Weights standardization equation. This equation considers the size of nodes, measured in terms of the number of papers associated with them. By translating this value into a scale ranging from 0 to 1, we were able to gauge the intensity of connections in relation to the specific context of current knowledge about both connected nodes. Edges weight, symbolized by edges thickness, represents the total number of papers in which a connected pattern was described between two nodes. These relationships were computed as standardized edges weight (_*st*_ *EW*) according to the following equation:

$$\text{stEW}=\left\{\begin{array}{c} \left(\frac{\alpha \times 100}{\beta }\right)/100,\;\text{if}\;\beta \geq \gamma \\ \left(\frac{\alpha \times 100}{\gamma }\right)/100,\;\text{if}\;\gamma \geq \beta \end{array}\right.,$$

where *α* represents the total edges weight measured by the total number of papers that simultaneously described two adaptive introgression characteristics; *β* represents the total number of papers that describe one of the characteristics; *ϒ* represents the total number of papers that describe the second characteristic.

The stEW metric was crafted for comparison purposes. Additionally, presenting the exact number of papers when assessing a link between two nodes aimed to offer supplementary information. Though not particularly handy for making comparisons, the number of papers gives valuable insight into the current understanding of a particular connection and nodes.

## 3 Results

### 3.1 The Temporal Evolution in the Trends in Knowledge on Adaptive Introgression

The number of publications on adaptive introgression has grown exponentially since 2012, driven by advances in genomic sequencing and computational technologies. This trend spans multiple fields, particularly evolutionary biology, with a marked increase in studies employing whole‐genome sequencing.

We recorded only a few studies published during the 20th century, with the highest prevalence in the 1990s, which saw an increase in published evidence for adaptive introgression, particularly from botanical controlled experimental studies involving the use of quantitative trait locus (QTL) (Figure S1—Supporting Information). A slight increase in the number of publications between 2000 and 2012 was observed alongside the rising of methodological developments from the Human Genome project (https://www.genome.gov/). However, more than 75% of the analyzed studies were published after 2012, when the annual average number of published papers increased from 1.9 ± 3.1 (1966–2011) to 34.5 ± 10.1 (2012–2019), equating to an average annual increase of 1824.14% of papers ($\chi_{1}^{2}$ = 97.37, *p* < 0.01).

This increased publication rate resulted from the wide application of genomic approaches, mainly through the advent of high throughput sequencing (HTS) and the use of whole‐genome sequencing and single nucleotide polymorphisms (SNPs) (Figure S1—Supporting Information). These techniques proved to be effective in detecting, in a cost‐effective way, genomic areas under introgression and their adaptive value. Increasing in an exponential way through time (GLM_SNPs [family = “poisson”]: *R* ^2^ = 0.80, *p* < 0.05, GLM_Whole‐Genome [family = “poisson”]: *R* ^2^ = 0.76, *p* < 0.05), these techniques also contributed to an exponential growth of adaptive introgression knowledge, which is evident from the cumulative number of published papers over time (GLM_Total Number of Papers [family = “poisson”]: *R* ^2^ = 0.94, *p* < 0.05) (Figure S1—Supporting Information). This growth pattern was evident in different scientific fields (Anthropology, Phylogeny/Genealogy, Plant Sciences, etc.), and particularly in Evolutionary Biology (Figure S1—Supporting Information).

**FIGURE 1:**
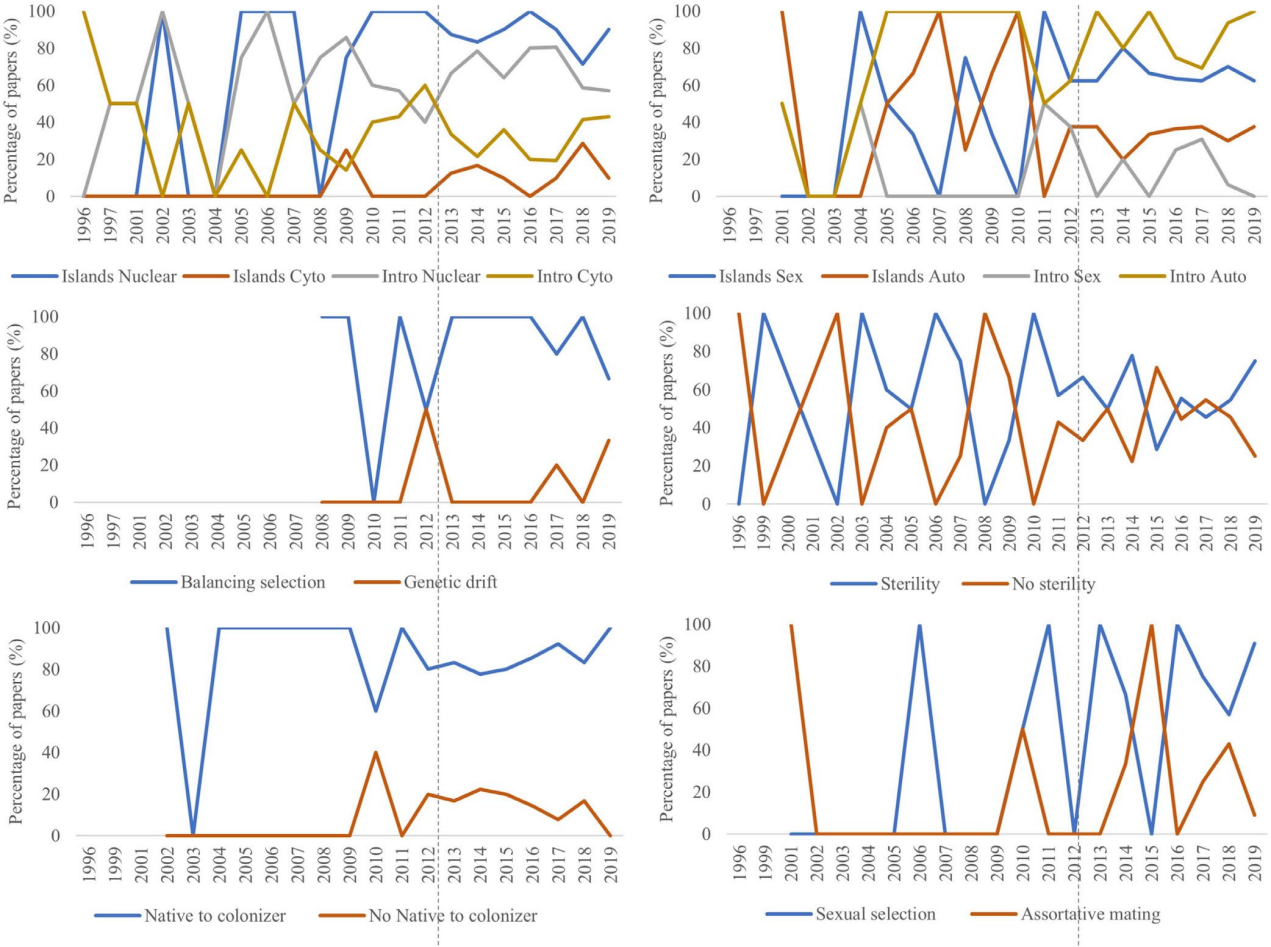
Temporal evolution of knowledge trends according to the percentage of papers published per year that provide evidence for specific characteristics of adaptive introgression. Dashed line shows the tipping point representing the beginning of the genomic revolution in adaptive introgression studies.

The genomic revolution allowed the establishment of a clearer understanding and stable patterns in the trends of knowledge, particularly about the genomic characteristics, their impacts on physiology, as well as the direction of adaptive introgression (Figure 1). These patterns encompassed: (i) a greater frequency of instances of autosomal nuclear introgression alongside islands of differentiation in sex‐linked chromosomes; (ii) a notably higher occurrence of balancing selection processes compared to genetic drift, as well as a prevailing trend of introgression from native to colonizer as opposed to the reverse direction; and (iii) a notable equilibrium, with approximately half of the annually published papers indicating some degree of sterility in the lineages involved in introgression, while the other half report an absence of sterility (Figure 1). In contrast, there were no clear patterns in behavioral aspects (Figure 1). Notably, after 2012, there was a considerable rise in evidence indicating the presence of divergent forces, such as islands of differentiation, genetic drift, or assortative mating, in the analyzed studies on adaptive introgression (Figure 1).

### 3.2 Structural Complexity of Organisms and Adaptive Introgression Characteristics

**FIGURE 2:**
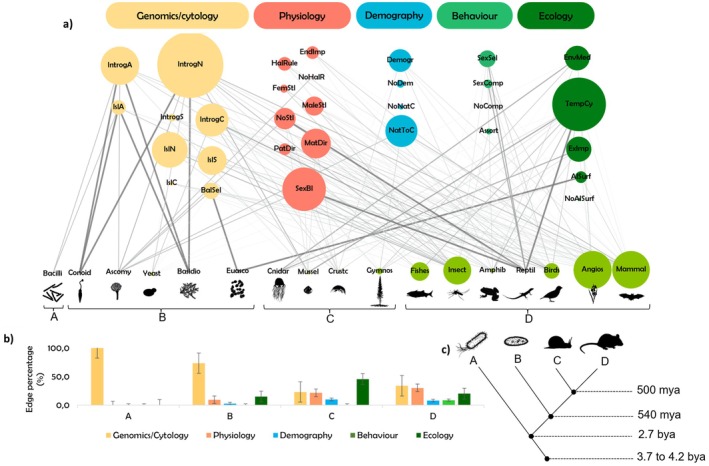
Bipartite network of adaptive introgression's reported characteristics, in different levels of biological organization, and the taxonomic groups, across a taxonomic complexity gradient (node size represents the total number of papers describing each characteristic and the edges' thickness represents standardized edges weights (stEW) of each connection (a). Bar chart showing the effect of the Taxonomic Group factor (A—Prokaryotes, B—Single‐cell eukaryotes, C—Eukaryotes with sex‐linked chromosomes and random mating, and D—Eukaryotes with sexual selection) on the percentage of connections across the levels of biological organization. Bars represent mean values with standard deviation. Significant results from detailed multiple comparisons of means are provided in Table S3 (Supporting Information) (b). Cladogram with the potential evolutionary cleavage moments of adaptive introgression functionality (c). Genomics/cytology's layer (Autosomal introgression [IntrogA], Autosomal islands of differentiation [IslA], Nuclear introgression [IntrogN], Introgression on Sex‐chromosomes [IntrogS], Cytoplasmic introgression [IntrogC], Islands of differentiation in nucleus [IslN], Islands of differentiation on Sex‐chromosomes [IslS], Cytoplasmic islands of differentiation [IslC], and the presence of balancing selection [BalSel]); Physiology's layer (endogenous impacts on adaptive introgression [EndImp], Haldane's rule compliance [HalRule], exception to Haldane's rule [NoHalR], female sterility [FemStl], male sterility [MalStl], any sterility [AnStl], sex‐bias introgression [SexBI], paternal biased‐direction of introgression [PatDir], and maternal biased‐direction of introgression [MatDir]); Demography's layer (demographic disparities [Demogr], any demographic disparities [NoDem], direction of introgression from native to colonizers [NatToC], and exception to native‐to‐colonizers direction [NoNatC]); Behavior's layer (presence of sexual selection [SexSel], presence of assortative mating [Assort], presence of intra‐sexual interspecific competition [SexComp], and absence of intra‐sexual interspecific competition [NoComp]); and Ecology's layer (environmental‐mediated introgression [EnvMed], iteration between allopatric and sympatric periods along evolutionary history [TempCy], exogenous impacts on adaptive introgression [ExImp], allele surfing [AlSurf], and no allele surfing [NoAlSf]).

Organismal complexity influenced patterns of adaptive introgression. Genomic‐level processes dominated in prokaryotes, while behavioral and ecological factors gained importance in more complex eukaryotes, particularly those with sexual selection mechanisms (Figure 2). Our meta‐analysis of trends in the published literature points to three main characteristics that seem to condition the described adaptive introgression: the eukaryotic cell, the presence of sex chromosomes, and the development of behavioral mechanisms of sexual selection (see Table 1—Glossary). Genomes/cytology covers almost half of the total connections between the taxonomic groups and adaptive introgression claims (46.7% ± 30.17%). Physiology comprises around 20% of connections (20.1 ± 16.88), followed by demographic aspects (6.3% ± 6.69%). Behavior represented just 3.5% ± 5.81% of total connections (Figure 2). The MANOVA revealed that the Taxonomic Group factor had a significant effect on the percentage of connections across the five levels of biological organization (Pillai's Trace = 1.390; *F*_(15,36)_ = 2.072; *p* = 0.037; Power = 0.887). Following the multivariate significance, Tukey's HSD post hoc test indicated that the significant differences were primarily observed in the genomics/cytology layer, with significantly higher connections in unicellular organisms and significantly lower connections in multicellular eukaryotes, regardless of their reproductive strategies. Notably, eukaryotes with sexual selection displayed significantly higher behavioral connections compared to all other groups (Table S3—Supporting Information).

In evolutionarily older and less complex organisms, namely in prokaryotes, adaptive introgression was addressed at the level of the genome, representing the only layer present in the majority of prokaryote networks (_st_EW = 0.11). For older single‐celled eukaryotic species, such as protists, genomes/cytology represented 73.3% ± 25.3% of all connections with their adaptive introgression studied characteristics. In contrast to prokaryotes, research on this group showed a clearer focus on cytological aspects of adaptive introgression, such as the positioning of introgression islands within cells (nucleus versus cytoplasm) (Figure 2).

Sexual reproduction in multicellular eukaryotic species with heteromorphic sex chromosomes brought new challenges to the investigation of adaptive introgression. For these more complex eukaryotes, studies demonstrated a pronounced emphasis on physiological aspects, aligning closely with the significance of genomes and cytology in terms of connection percentage (21.5% ± 18.3%, _st_EW_[physiology]_ = 0.07; 23.0% ± 19.5%, _st_EW_[genomes/cytology]_ = 0.08, respectively), particularly in studies on crustaceans (44.4%, _st_EW = 0.15), mussels (24.0%, _st_EW = 0.10) and gymnosperms (17.7%, _st_EW = 0.05). In these older multicellular eukaryotes with differing sex chromosomes, adaptive introgression was frequently influenced by demographic factors. Once reproduction becomes stochastic in contact zones, demographic asymmetries were shown in the literature to become a fundamental mechanism for promoting directional introgression from the most abundant to the least abundant species. Gymnosperms were probably the most prominent example of this (20.6%, _st_EW = 0.13). In contrast, in more recent and complex eukaryote species like birds or mammals (genomically and behaviorally), adaptive introgression studies tend to address issues related to sexual behavior (Figure 2). Eukaryotes with mating preferences were the only ones whose behavioral characteristics were shown to influence the likelihood of successful adaptive introgression (8.4% ± 6.4%, _st_EW = 0.15). This pattern was particularly clear in birds and reptiles' studies (12.1%, _st_EW = 0.21 and 21.5%, _st_EW = 0.33, respectively).

Finally, research consistently highlighted the influence of ecological factors (e.g., exposure to pesticides, changes in temperature) on adaptive introgression, irrespective of the species' taxonomic complexity, from single‐celled eukaryotes (15.0% ± 22.4%, _st_EW = 0.06), older eukaryotes with sex‐linked chromosomes and stochastic breeding choices (45.5% ± 39.2%, _st_EW = 0.22) to eukaryotes capable of performing sexual selection (20.1% ± 8.2%, _st_EW = 0.18) (Figure 1).

### 3.3 The Known Evolutionary Mechanisms and Characteristics of Adaptive Introgression

Adaptive introgression in eukaryotic species predominantly occurs in nuclear DNA, often driven by balancing selection and, apparently, independent of the timing of introgression events. In species with sex‐linked chromosomes, where introgression is less frequent, it often aligns with Haldane's rule, typically exhibiting sex‐biased patterns associated with sterility or unviability. Random mate choice or sexual selection influences directional introgression, with demographic asymmetries often favoring native‐to‐colonizer gene flow and shaping sex‐biased dynamics. Environmental factors, both natural and anthropogenic, also play a critical role, often mediated by historical cycles of divergence.

#### 3.3.1 Adaptive Introgression in Eukaryotic Species

**FIGURE 3:**
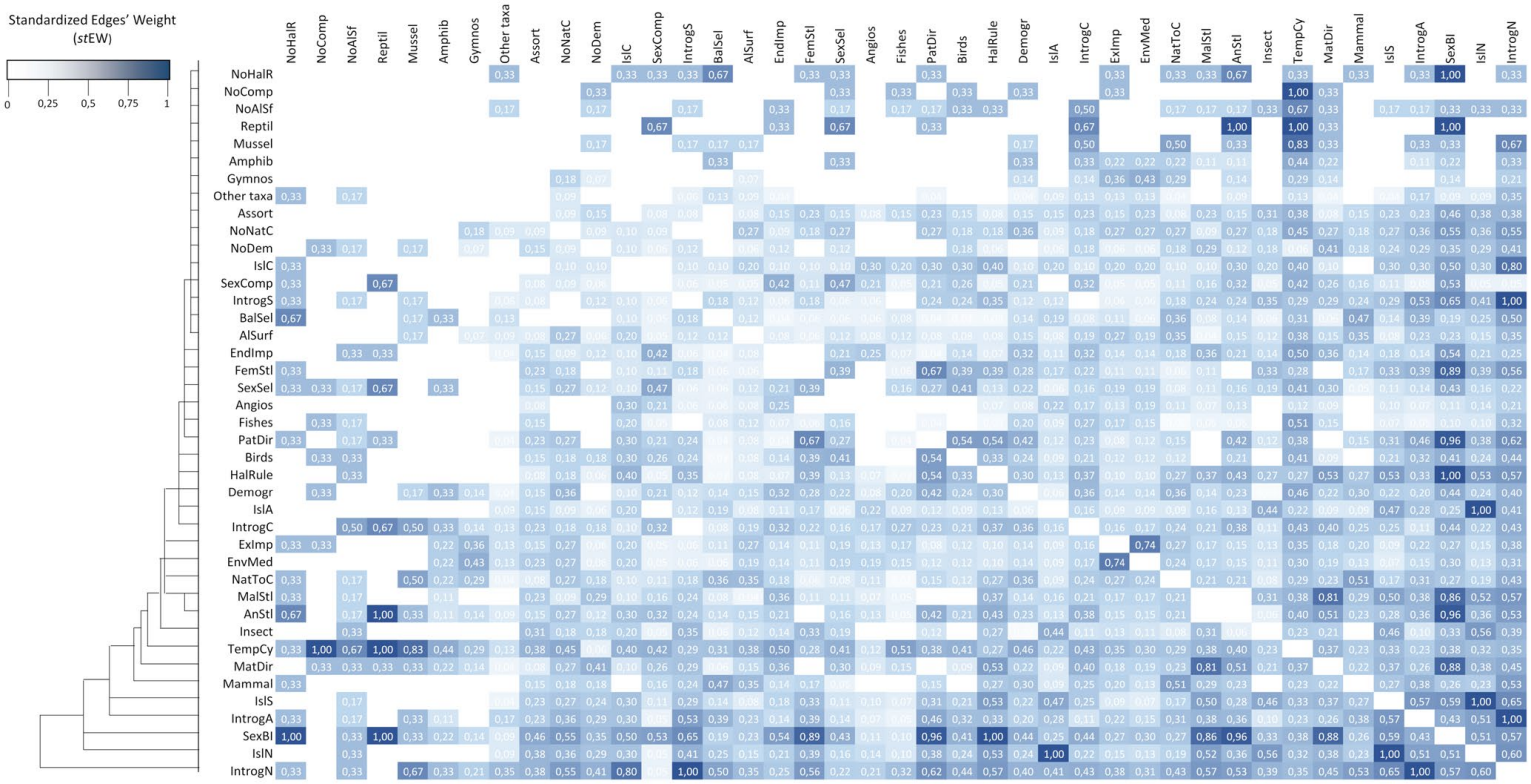
Heatmap of pairwise standardized edges' weight (_st_EW) among adaptive introgressions reported characteristics and the taxonomic groups.

**FIGURE 4:**
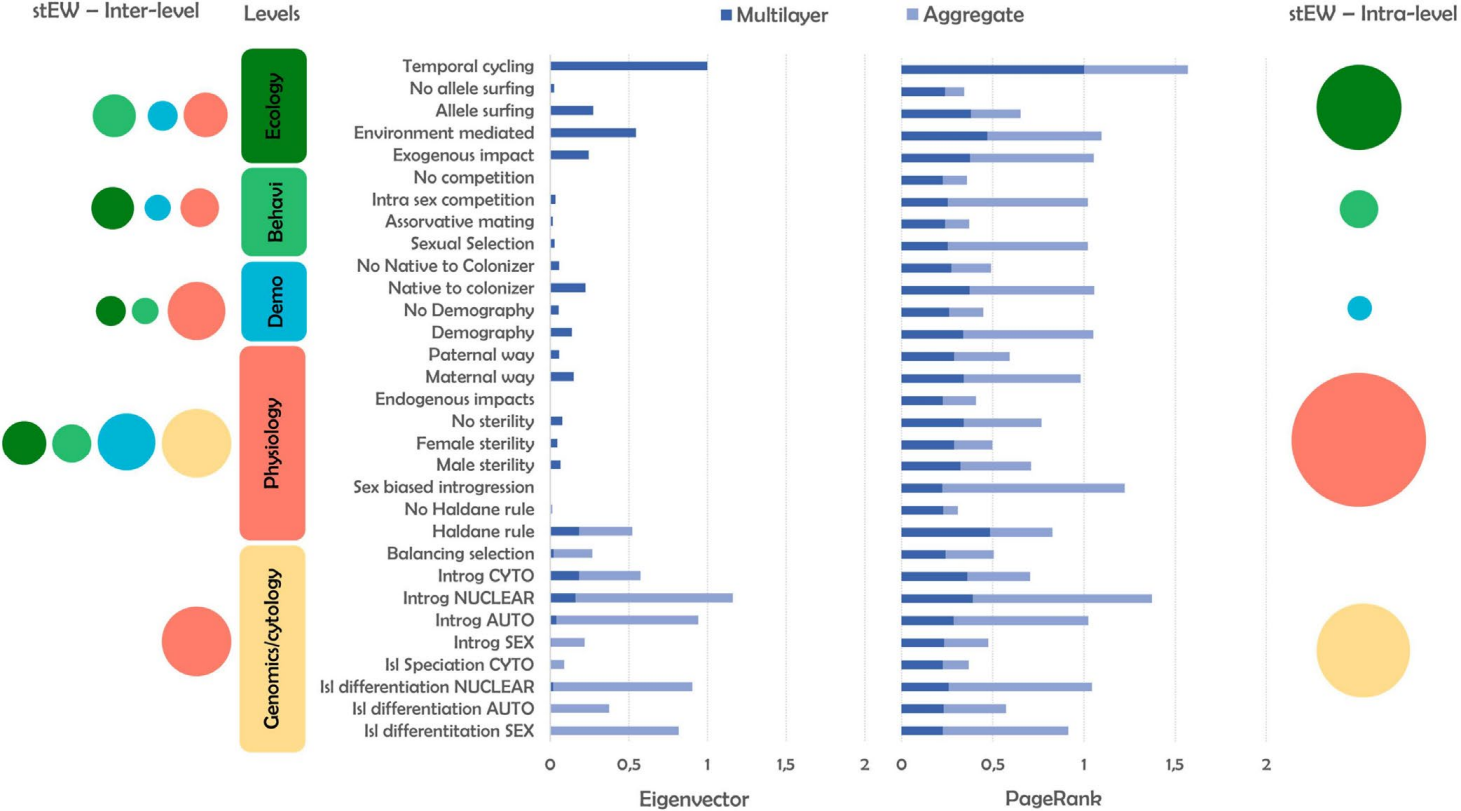
Eigenvector and PageRank metrics from multilayer network analyses considering both multilayer values (across different levels) and aggregate values (without level consideration). Circles size represents the average of standardized edges weights (stEW) between different adaptive introgression characteristics at inter‐level (left side) and intra‐level (right side).

We found studies showcasing evidence of introgressed alleles with adaptive effects in both cytoplasmic DNA (cDNA) and nuclear DNA (nDNA), either exclusively or concurrently (_st_EW = 0.43, *N* = 31 papers) (Figure 3), albeit several cases of genetic incompatibilities, including cytonuclear discordance (stEW = 0.38, *N* = 12) (Gagnaire et al. 2012). However, many described cases showed lower levels (or even none) of cytoplasmic introgression, particularly when compared to nuclear introgression (Brown et al. 2019; Carling and Brumfield 2008; Hulsey et al. 2013; Morales et al. 2017). Our results showed that nuclear introgression is not only the most frequently reported type of introgressed gene transfer in eukaryotic genomes but also the most prevalent characteristic of introgression across all published studies (Eigenvector = 1.00, PageRank = 0.98, *N* = 144 paper) (Figure 4). However, we found no association between the timing of introgression (ancient versus current events) and the genomic location of introgression (cDNA versus nDNA) ($\chi_{1}^{2}$ = 3.46; *p* > 0.05). Balancing selection was more prevalent in adaptive introgression studies than genetic drift (Eigenvector = 0.11, PageRank = 0.26, *N* = 32 papers) ($\chi_{1}^{2}$ = 13.23; *p* < 0.01), impacting mainly nuclear introgression (_st_EW = 0.50, *N* = 18 papers) (Figure 3).

#### 3.3.2 Adaptive Introgression in Species With Sex‐Linked Chromosomes

Our results demonstrated that there are fewer reported cases of adaptive introgression in cDNA (Eigenvector = 0.40, PageRank = 0.34, *N* = 72 paper) than into autosomes (Eigenvector = 0.90, PageRank = 0.74, *N* = 84 papers) (Carling and Brumfield 2008). However, they were both much more commonly reported than those in sex‐linked chromosomes (Eigenvector = 0.21, PageRank = 0.24, *N* = 17 papers). Introgression in sex‐linked chromosomes was never simultaneously described with cDNA introgression, occurring just in parallel with autosomal introgression (_st_EW = 0.53, *N* = 9 papers) (Figure 3). Species with heteromorphic sex chromosomes, in particular mammals, showed lower rates of introgression in sex‐linked chromosomes compared to autosomes ($\chi_{2}^{2}$ = 19.14; *p* < 0.01).

Several studies, covering numerous taxa, have simultaneously identified islands of differentiation primarily located in sex‐linked chromosomes, as well as instances of autosomal introgression (_st_EW = 0.57, *N* = 36 papers; Figure 3). Nevertheless, there were important exceptions in the genomic location of islands of differentiation (Autosomes: Eigenvector = 0.37, PageRank = 0.34, *N* = 32). Only one paper provided evidence for either cytoplasmic or autosomal islands of differentiation and genetic drift (_st_EW = 0.13).

We found papers with evidence for islands of differentiation in sex‐linked chromosomes alongside some degree of sex sterility/unviability in 28 papers (_st_EW = 0.51), which was particularly high compared to non‐sterility cases (_st_EW = 0.28, *N* = 13 papers) ($\chi_{1}^{2}$ = 4.23; *p* < 0.05). Both islands of differentiation in sex‐linked chromosomes and sterility/unviability should affect preferentially the heterogametic sex (_st_EW = 0.53, *N* = 16 papers and _st_EW = 0.60, *N* = 18 papers, respectively) compared to the homogametic sex (_st_EW = 0.00, no papers and _st_EW = 0.33, two papers, respectively) (Figure 3).

In line with Haldane's rule (Haldane 1922; Kulmuni and Pamilo 2014), the sex with the heteromorphic sex chromosome showed greater hybrid fitness reduction (Eigenvector = 0.40, PageRank = 0.34, *N* = 30 papers) through either increasing sterility (completely or partially) or even their unviability (see Table 1—Glossary) (Kulmuni and Pamilo 2014). A significantly higher number of cases complied with Haldane's rule than those that did not ($\chi_{1}^{2}$ = 20.48; *p* < 0.01). The applicability of Haldane's rule seems to stem indirectly from the presence of islands of differentiation in the sex chromosomes (_st_EW = 0.53, *N* = 16). However, in several cases that corroborate Haldane's rule, sterility or unviability was not reported (_st_EW = 0.43, *N* = 13 papers), or papers provided evidence that islands of differentiation do not always result in sterility or unviability (_st_EW = 0.28, *N* = 13).

Sex‐biased sterility/unviability (_st_EW_[males]_ = 0.86, *N* = 36 papers; _st_EW_[females]_ = 0.89, *N* = 16 papers) and endogenous impacts on reproductive cells and/or secondary sexual characteristics (_st_EW = 0.54, *N* = 15 papers) can both promote sex‐biased introgression (Walsh et al. 2016). Sex‐biased introgression is one of the most important introgression characteristics among all published studies (Eigenvector = 0.59, PageRank = 1.00, *N* = 95; Figure 4). Our results showed that a large number of papers in compliance with Haldane's rule (_st_EW = 1.00, *N* = 30) were a fundamental outcome of the presence of islands of differentiation in sex‐linked chromosomes (stEW = 0.59, *N* = 37). On the other hand, high co‐occurrence with cases of introgression in sex‐linked chromosomes revealed that Haldane's rule also contributed to introducing sex bias in the introgression process (_st_EW = 0.65, *N* = 11 papers), which likely did not occur through the directly promoting sex‐biased sterility (or even total unviability) (_st_EW = 0.24, *N* = 4 papers).

Our results also showed high connectivity in the literature between sex‐biased introgression and exceptions to Haldane's rule (_st_EW = 1.00, *N* = 3 papers), as well as cases of absence of sterility/unviability (_st_EW = 0.96, *N* = 45 papers). Female‐mediated introgression (Eigenvector = 0.46, PageRank = 0.64, *N* = 65 papers) was common among adaptive introgression case studies due to some degree of male sterility/unviability (_st_EW = 0.81, *N* = 34 papers) in cases where they are the heterogametic sex (particularly in mammals [_st_EW = 0.22, *N* = 14 papers] and insects [_st_EW = 0.21, *N* = 13 papers]), following the assumptions of Haldane's rule (_st_EW = 0.53, *N* = 16 papers). Despite male‐mediated introgression being significantly less reported (Eigenvector = 0.18, PageRank = 0.30, *N* = 26 papers) ($\chi_{1}^{2}$ = 15.87; *p* < 0.01), it follows the same pattern as a result of sex‐biased introgression (_st_EW = 0.96, *N* = 25 papers). This pattern is closely related to high levels of female sterility/unviability (_st_EW = 0.67, *N* = 12 papers) in rarer cases in which they are the heterogametic sex (particularly in birds [_st_EW = 0.54, *N* = 14 papers]).

#### 3.3.3 Adaptive Introgression in Species Under Random Mate Choice or Sexual Selection

Demographic asymmetries can significantly impact how adaptive introgression unfolds in species complexes where mate selection between two interbreeding species involves some degree of randomness. (Eigenvector = 0.27, PageRank = 0.71, *N* = 50 papers). In those cases, adaptive introgression is more frequently reported to be directed towards the less abundant species (Eigenvector = 0.08, PageRank = 0.19, *N* = 17 papers) ($\chi_{1}^{2}$ = 15.28; *p* < 0.01). In line with this pattern, which is often connected to demography or stochastic mate choices (_st_EW = 0.36, *N* = 18 papers), we found the asymmetric direction of adaptive introgression from native‐to‐colonizer species is one of the most cited introgression patterns (Eigenvector = 0.28, PageRank = 0.68, *N* = 70 papers). Native‐to‐colonizer direction is much more frequently reported than the rarer examples of the reverse directional introgression from colonizer‐to‐native species (Eigenvector = 0.07, PageRank = 0.22, *N* = 11 papers) (Figure 4).

Balancing selection processes seem to be strictly related to this native‐to‐colonizer demographic pattern (_st_EW = 0.36, *N* = 13 papers). This direction of introgression is a pattern that seems to affect mainly older taxa (e.g., _st_EW_[Mussels]_ = 0.50, *N* = 3 papers; _st_EW_[Gymnosperms]_ = 0.29, *N* = 4 papers). However, high values of published evidence of adaptive introgression from native‐to‐colonizer in mammal species (_st_EW = 0.51, *N* = 36 papers) suggest the influence of other phenomena than demographic asymmetries in species abundances. We found a possible relationship between examples of some degree of random mate choice and the opposite direction of adaptive introgression from colonizers‐to‐native species (_st_EW = 0.36, *N* = 4 papers) (Figure 3).

Sexual selection, defined as a breeding behavior favoring mating partners with a fitness advantage independent of their species (Eigenvector = 0.19, PageRank = 0.77, *N* = 37 papers), showed fewer reported cases of co‐occurrence with demographic asymmetries (_st_EW = 0.22, *N* = 8 papers). Sexual selection pieces of evidence show a high rate of connectivity with the cases of sex‐biased introgression (_st_EW = 0.43, *N* = 16 papers), primarily by impacting female or male breeding choices, depending on whether the introgression case study is female‐or male‐mediated (_st_EW = 0.30, *N* = 11 papers and _st_EW = 0.27, *N* = 7 papers, respectively). This phenomenon was more prevalent in the literature than assortative mating, which is an opposite breeding force in a conspecific direction (Eigenvector = 0.06, PageRank = 0.16, *N* = 13 papers) ($\chi_{1}^{2}$ = 10.58; *p* < 0.01). However, there are important examples of the occurrence of assortative mating at the same time as adaptive introgression (Eigenvector = 0.06, PageRank = 0.13, *N* = 13 papers). It could happen even in cases where sexual selection appears as an important force determining the direction of adaptive introgression (_st_EW = 0.15, *N* = 2 papers) (Figure 3).

Intra‐sexual interspecific competition (Eigenvector = 0.12, PageRank = 0.77, *N* = 19 papers) is another mating behavior reported to contribute to directional introgression, with published evidence showing high levels of co‐occurrence with sexual selection (_st_EW = 0.47, *N* = 9 papers). This association illustrates that both sexes can have fundamental roles in sexual selection: one sex may select the fittest partner while the other competes actively against individuals of other species for reproductive opportunities. More frequently, studies described the presence of intra‐sexual competition compared to its absence ($\chi_{1}^{2}$ = 10.23; *p* < 0.01), being strongly connected with case studies demonstrating sex‐biased introgression (_st_EW = 0.53, *N* = 10 papers). The analyzed literature showed a co‐occurrence between this type of competition and endogenous factors that impact the direction of adaptive introgression (_st_EW = 0.42, *N* = 8 papers) (Figure 3).

#### 3.3.4 Evidence of Environmental Impacts on Adaptive Introgression

Several papers showed examples of exogenous factors exerting influences over the adaptive introgression process (Eigenvector = 0.26, PageRank = 0.68, *N* = 55 papers), mainly through natural environmental factors (_st_EW = 0.74, *N* = 40 papers) despite the very relevant examples of adaptive introgression in response to anthropogenic impacts ($\chi_{1}^{2}$ = 17.36; *p* < 0.01). On the other hand, regarding the geographical extent of adaptive introgression, there are still few empirical examples of introgressed alleles outside contact zones through allele surfing (Eigenvector = 0.11, PageRank = 0.27, *N* = 26 papers) (Figure 4). Finally, the historical iteration between different periods of divergence and adaptive introgression was one of the most relevant described patterns in the case studies (Eigenvector = 0.40, PageRank = 0.57, *N* = 117 papers) frequently mediated by fluctuations in environmental conditions (_st_EW = 0.35, *N* = 19 papers) (Figure 3).

## 4 Discussion

### 4.1 Main Findings

Our study underscores the central role of adaptive introgression as a key evolutionary force in synthesizing knowledge across diverse taxa and biological dimensions (Pfennig 2021). Key findings highlight the predominance of nuclear autosomal introgression, often accompanied by islands of differentiation in sex‐linked chromosomes, as shown in Figures 3 and 4. Our results notably reveal a greater influence of balancing selection over genetic drift, particularly affecting nuclear introgression, with many studies identifying balancing selection as a key driver of adaptive introgression. Introgression patterns often display a directional bias from native to colonizing species, in alignment with Haldane's rule, and typically show sex‐biased patterns related to sterility or unviability. The meta‐analysis identifies three key organismal characteristics shaping adaptive introgression: the eukaryotic cell, sex‐linked chromosomes, and sexual selection. The complexity of adaptive introgression traits increases across the tree of life, with bacteria exhibiting less complex interactions compared to mammals. While introgression operates at the genomic level, its complexity spans multiple biological levels, impacting cytological features, as well as physiological, demographic, and behavioral dimensions. Our findings suggest that adaptive introgression operates within a broader evolutionary context, where divergence can coexist with gene flow, often mediated by both natural and anthropogenic environmental factors.

### 4.2 Scope and Evolution of Knowledge About Adaptive Introgression

Our study supports the growing evidence of a shift in understanding adaptive introgression, particularly since the genomic revolution of the early 2010s. The rise of high‐throughput sequencing technologies, like whole‐genome sequencing and RADseq, allowed researchers to identify genomic regions under selection more effectively. This, combined with a significant decline in sequencing costs, expanded evidence for adaptive introgression across a broader taxonomic and geographic range, helping clarify its genomic characteristics, physiological impacts, and evolutionary role (Hayden 2014; Kuhlwilm et al. 2019). In line with these advancements, our findings confirm key trends that emerged post‐2012, including the predominance of nuclear autosomal introgression, often coupled with islands of differentiation in sex‐linked chromosomes. Additionally, our analysis shows a higher prevalence of balancing selection over genetic drift, which aligns with observations from several post‐genomic revolution studies (Nadachowska‐Brzyska et al. 2012; While et al. 2015). We also found a clear directional bias in introgression patterns, favoring introgression from native to colonizer species, a trend consistent with earlier work on Haldane's rule and sex‐biased introgression patterns, as discussed in studies like Mallet (2005) and Feder et al. (2012). This bias was particularly evident in species that show sterility or unviability in hybrids, which aligns with our findings of sex‐biased introgression in several taxa.

The increasing recognition of the evolutionary advantages provided by genetic admixture has reshaped perceptions of introgression, moving beyond historical biases that often downplayed its significance (Arnold et al. 2012). Our results reflect this shift, as we see a growing acknowledgment of introgression's role in facilitating adaptive evolution. This has significant implications for societal and policy discussions (Baker 2016; Fredrickson 2002) and for applied fields like conservation biology, particularly in shaping strategies to preserve adaptive diversity (Biermann and Mansfield 2014; Chan et al. 2019). Similarly, the agriculture and animal husbandry industries have been exploring this topic to leverage insights into introgression's potential for enhancing adaptive traits (Henkel et al. 2019).

Our meta‐analysis also highlights that post‐2012 studies have provided stronger evidence that speciation can occur even in the presence of gene flow. This finding is consistent with the growing body of literature suggesting that hybridization and introgression can promote adaptation without necessarily hindering speciation. This concept was foundational in early studies (Mayr 1942) and has been further developed in recent years (Harrison and Larson 2014; Mallet 2005), illustrating that the two processes are not mutually exclusive, even when their evolutionary forces act in opposing directions, namely convergence and divergence.

The methodological and technological advances of the past two decades have been the primary drivers of the significant expansion in adaptive introgression research. Our study highlights its current influence in the growing recognition of adaptive introgression as a critical evolutionary force, broadening its scope by integrating genomic, physiological, ecological, and behavioral dimensions into a more comprehensive understanding of adaptation. By analyzing a diverse range of species, from prokaryotes to mammals, we demonstrate that adaptive introgression is not limited to a few model organisms but is instead a widespread phenomenon with profound implications for evolutionary biology.

### 4.3 Structural Complexity of Organisms Impacting Adaptive Introgression Characteristics

Our results show that in prokaryote species, adaptive introgression operates primarily through genetic recombination via chromosomal conjugative mobilization, aligning with previous studies (McAshan et al. 1999) and underscoring its importance in providing adaptive advantages, such as antibiotic resistance (see McAshan et al. 1999; Palmer et al. 2010). However, plasmids comprise a substantial fraction of the auxiliary genome, being responsible for much of their natural gene transfer (Palmer et al. 2010). The prevalence of balancing selection observed in our meta‐analysis suggests that processes of selection, rather than genetic drift, play a key role in the fixation of introgressed alleles, particularly in prokaryotic species, counteracting the random processes that may cause rare alleles to disappear in a few generations. However, the higher occurrence of balancing selection cases observed in the literature (Nadachowska‐Brzyska et al. 2012; While et al. 2015) might be attributed to an emphasis on genes that potentially convey adaptive advantages, thereby mitigating the impact of genetic drift. Conversely, introgression events lost due to genetic drift are often challenging to quantify simply because they have vanished.

In eukaryotic species, our findings indicate that adaptive introgression is strongly influenced by cytonuclear discordance, differences in recombination rates, and selection within the cell. This supports the conclusion from our meta‐analyses that introgression primarily occurs in the nucleus, especially in autosomes, likely in regions not strictly associated with cDNA or not resistant to recombination, and is often driven by selection pressures at the cellular level (Burton and Barreto 2012; Derr et al. 2012; Matosiuk et al. 2014).

In multicellular eukaryotic species with heteromorphic sex chromosomes, while our study shows that adaptive introgression occurs mainly in autosomes, islands of differentiation are fundamentally present in sex‐linked chromosomes. Sex‐linked chromosomes are involved in specific functions in reproduction that exert pressures, for example, over breeding physiology. Thus, introgression in sex chromosomes can sometimes have negative consequences like sex‐biased sterility or even unviability (Storchová et al. 2004). However, a high prevalence of studies identifying sex‐biased introgression indicates organisms can bypass these physiological constraints (i.e., sex‐biased sterility or even unviability).

The literature indicates that demographic discrepancies, such as differences in species abundances, drive mating toward the most abundant species, particularly in cases of random mate choice. It has been argued that in extreme cases it could even lead to the disappearance of the rarest species through genetic swamping (Todesco et al. 2016). However, when adaptive introgression is asymmetrical and directionally biased toward the less abundant species (a particular directional pattern that arises from our meta‐analysis), it may allow this species to incorporate advantageous traits from the more abundant species. This is a potential evolutionary process that enables the rarer species to adapt to the environment, overcoming the constraints of both outbreeding depression and genetic swamping.

In more recently evolved and complex eukaryote species, sexual behaviors, such as non‐random mating, can lead to assortative mating, whereby individuals tend to seek conspecific mating partners, reducing the occurrence of introgression (Hume et al. 2018). However, our results show that when mating is not random, there is a tendency to favor the fittest individuals through sexual selection, independently of the species (Parrett and Knell 2018). This behavior may allow the less fit species to adapt and thrive by incorporating the traits that give its sister species higher fitness. It could help to explain how these species can bypass restrictions resulting from assortative mating.

### 4.4 Known Evolutionary Mechanisms and Characteristics of Adaptive Introgression

As organisms evolve and increase in structural complexity, new challenges to adaptive introgression arise from barriers to gene flow, including genetic changes (e.g., inversions, fusions), innovations in cellular and physiological systems, and shifts in mating strategies. Nevertheless, adaptive introgression can still occur. Evolutionary forces resulting from divergence, which drive distinct adaptations in different populations, do not necessarily preclude introgression. Instead, processes such as population diversification and gene exchange often coexist and interact, fostering adaptation in dynamic and interconnected ways. This dynamic interplay is further explored in the following sections, which examine the mechanisms that allow adaptive introgression to persist despite opposing evolutionary forces.

#### 4.4.1 Balancing Selection Versus Genetic Drift

The probability that introgressed genes will become established in the genomes of the receptor taxa is still under debate. Because these genes are initially rare, they are more likely to be particularly sensitive to stochastic processes such as genetic drift. This effect is particularly pronounced in populations where sexual selection is weak or absent, and when effective population sizes are low (Gompert et al. 2013).

However, while genetic drift can lead to the loss of introgressed alleles, balancing selection promotes adaptive introgression by maintaining multiple alleles at higher frequencies, counteracting processes like selection for conspecific breeding partners (assortative mating) or self‐fertilization (King et al. 2006). Our findings emphasize that balancing selection often underpins adaptive introgression, reducing allele loss through drift and supporting the establishment of adaptive alleles. The higher occurrence of balancing selection suggests that introgressed alleles are more likely to be maintained within populations, while genetic drift may limit their persistence in smaller populations. Thus, balancing selection does not merely reduce allele loss due to drift, but rather actively contributes to the establishment of adaptive introgression (While et al. 2015). Not all mechanisms of balancing selection, however, are likely to promote adaptive introgression in the same way (see While et al. (2015) for further discussion). For instance, genes under negative frequency‐dependent selection, which have higher migration rates, may allow for more efficient introgression and establishment in the gene pool of the receptor species (Brennan et al. 2013). This mechanism supports our findings that adaptive introgression is more likely to occur when balancing selection operates in conjunction with gene flow.

#### 4.4.2 Autosomal Introgression Versus Cytonuclear Discordance

Structural variations such as chromosomal inversions, translocations, and fusions can contribute to genetic incompatibilities, including cytonuclear discordance. These structural changes can disrupt recombination and create barriers to gene flow, promoting reproductive isolation and divergence. Specifically, chromosomal inversions can suppress recombination in hybrid zones, preserving incompatible allele combinations or cytonuclear mismatches, which can lead to reduced fitness in hybrids. Our review supports these assumptions by identifying a consistent set of studies that highlight phenomena such as chromosomal inversions often harboring co‐adapted gene complexes, preventing recombination with incompatible alleles from other populations (see Hoffmann and Rieseberg 2008; Feder et al. 2012). This preservation can stabilize genetic incompatibilities that may involve interactions between nuclear and mitochondrial genes, especially in the presence of epistatic relationships or underdominance (Feder et al. 2012). Additionally, chromosomal rearrangements may enhance the likelihood of such incompatibilities by altering gene expression or disrupting regulatory networks crucial for cytonuclear interactions (Navarro and Barton 2003).

Cytonuclear discordance can impact species' fitness, leading to reduced fertility and/or disease, or even promoting the formation of unviable hybrids (Burton and Barreto 2012; Derr et al. 2012; Matosiuk et al. 2014). Despite some reported cases of genetic incompatibilities, including cytonuclear discordance (see Gagnaire et al. 2012), our results emphasize the occurrence of introgression in both cDNA and nDNA in co‐introgression, indicating that individuals with the nuclear genome of one species (in full or in part) and the cytogenome of another (in full or in part) are frequently viable and able to backcross (Pujolar et al. 2014; Sarver et al. 2017).

The location of introgression in the genome of eukaryotes is associated with cytonuclear discordances, a phenomenon more commonly observed in animal studies than in the plant literature (Morales et al. 2018). The majority of reported adaptive introgression cases in our review occur at the nuclear level (Hulsey et al. 2013), although some authors consider that organelle genomes introgress more easily and more frequently than nuclear genomes (Giannoulis et al. 2019; Hawkins et al. 2016; Wade and Goodnight 2006). However, our findings support that, while nuclear introgression is responsible for cytonuclear discordance only under a narrow range of conditions (Bonnet et al. 2017), strong discordance is often driven by cytoplasmic genome selection, particularly in biological systems with limited dispersal capacity (Brower 2018).

Our findings support the idea that cDNA introgression can occur without promoting discordance, mainly if it is unconnected to nDNA regions responsible for interspecific incompatibilities (Di Candia and Routman 2007). cDNA single‐parent inheritance also reinforces the importance of sex‐biased dispersal (Kulikova et al. 2004) for its adaptive introgression, impacting cytonuclear gene interactions (Llopart et al. 2014). Nevertheless, after a period of isolation, cytonuclear interactions may evolve separately (Herrig et al. 2014). Therefore, disequilibrium between cDNA and autosomal haplotypes (Oliver et al. 2002) can minimize the likelihood of cDNA introgression (Herrig et al. 2014), making nDNA introgression with no cDNA gene flow a common phenomenon among adaptive introgression case studies, which may explain the pattern emerging from our results.

#### 4.4.3 Autosomal Introgression Versus Islands of Differentiation in Sex‐Linked Chromosomes

Nuclear asymmetric introgression, favoring autosomes (see Carling and Brumfield 2008), means sex chromosomes become the main candidate for forming barriers to gene flow and storing islands of differentiation (Runemark et al. 2018). Our results confirm this pattern, showing that introgression predominantly occurs in autosomes, while islands of differentiation are more commonly found in sex‐linked chromosomes. Thus, the concurrent presence of islands of differentiation in sex chromosomes alongside adaptive autosomal introgression was identified as a prevalent pattern in the literature. This often represents an equilibrium between divergent selection and genetic homogenization forces within the nucleus (Minder and Widmer 2008). However, islands of differentiation—defined as genomic regions exhibiting unusually high levels of differentiation between populations or species (Nachman and Payseur 2012)—can be generated by processes unrelated to speciation (Nosil et al. 2012), as in the case of reduced diversity due to linked selection (Cruickshank and Hahn 2014). Differences in recombination rates in sex chromosomes may help to explain the high centrality of islands of differentiation in sex‐linked chromosomes in our meta‐analysis (Wadsworth et al. 2015). Sex chromosomes have characteristics, such as morphological and genetic differentiation between the sexual chromosomes X and Y, or Z and W, or restrictions to recombination (Storchová et al. 2004). These characteristics together with the fundamental role they play in sexual reproduction (e.g., formation of gametes) make them particularly resistant to introgression in species with dioecious sexual reproduction. Our analysis shows that sex‐linked chromosomes are often resistant to introgression due to these constraints, yet when introgression occurs, it frequently involves regions with functional relevance to reproduction. Limitations in the variation of DNA repair and genes with mitochondrial function (Runemark et al. 2018) and demographic aspects derived from the single‐parent inheritance of sex chromosomes (in the heterogametic sex), (e.g., sex‐biased dispersal) (see Kulikova et al. 2004), can explain the rarity or even absence of introgression in sex chromosomes (Cahill et al. 2015), a similar process that happens with haplodiploid species like ants and bees (see Ghenu et al. 2018). These findings are consistent with our results, where introgression in sex chromosomes was observed less frequently than in autosomes but showed clear functional impacts when it occurred. This may be explained by the unique exposure of sex chromosomes, particularly in the heterogametic sex, to selection pressures, which can enhance their adaptive evolution capacity despite gene loss in non‐recombining chromosomes (e.g., Y chromosomes) (Muirhead and Presgraves 2016).

The presence of islands of differentiation on sex‐linked chromosomes does not always lead to divergence, as seen in genomic regions with reduced diversity, helping to explain why they do not always result in sterility or unviability (Cruickshank and Hahn 2014). Our meta‐analysis supports this, showing that islands of differentiation in sex chromosomes often coexist with gene flow in autosomes, maintaining a balance between selection and converging forces. Exceptions in the genomic location of islands of differentiation, whether on autosomes or cDNA (Sun et al. 2012), do not contain genes that influence reproductive isolation but have still undergone significant differentiation, often as a result of random processes, such as genetic drift (Nosil et al. 2012).

#### 4.4.4 Sex‐Biased Introgression Versus Physiological Constraints

Islands of differentiation in sex‐linked chromosomes are typically only a few hundred kilobases in size, whereas introgressed regions can extend over several megabases (Gagnaire et al. 2013). This asymmetry arises from the faster evolution and meiotic drive of sex chromosomes (Sun et al. 2012). Islands of differentiation in sex‐linked chromosomes may act like breeding barriers, being related to temporal and behavioral isolation (Wadsworth et al. 2015), namely affecting individuals' sex‐biased sterility/unviability (see Dopman et al. 2005). Both islands of differentiation in sex‐linked chromosomes and sterility/unviability show high connectance according to our results and affect preferentially the heterogametic sex. This aligns with Haldane's rule, as highlighted in our results, where heterogametic individuals experience greater fitness reductions (see Haldane 1922; Kulmuni and Pamilo 2014), often mediating asymmetric introgression patterns. The reviewed literature corroborates that physiologic outcomes deriving from Haldane's rule are frequently responsible for promoting asymmetric patterns of sex‐biased introgression (Sackton et al. 2011). These patterns are commonly mediated by females, especially in cases of homogametic males with some level of postzygotic breakdown. Our analysis identified examples of sex‐biased introgression where Haldane's rule was upheld, with introgressed alleles more frequently maintained in homogametic males due to selective pressures favoring reproductive viability. Examples of simultaneous occurrences between sex‐biased introgression and exceptions to Haldane's rule are also described in the literature, probably related to demographic and/or behavioral traits, such as positive selective heterosis in a single sex (uniparental introgression) (Beresford et al. 2017). Moreover, the sex with the higher dispersal rate usually presents higher levels of gene flow with conspecific populations outside the contact zone (Kulikova et al. 2004; Morales et al. 2018). On the other hand, though rarely identified in our literature review, islands of differentiation and/or introgression in sex‐linked chromosomes can also express other endogenous impacts on reproductive cells and on secondary sexual characteristics, which can also lead to a decrease in hybrid and backcross fitness (Walsh et al. 2016).

#### 4.4.5 Directional Adaptive Introgression Versus Genetic Swamping

Stochastic processes, such as colonisations, extinctions, and bottlenecks, can influence species survival (Vaillant et al. 2013). The literature reviewed supports this fact, showing that adaptive introgression can occur in contexts where stochastic events reshape population dynamics, such as during colonization or bottlenecks (Lexer et al. 2004). When species differ in abundance, mating tends to favor the more abundant species (Lexer et al. 2010). This typically results in gene flow from the dominant species to the rarer one, potentially leading to its disappearance through genetic swamping (Lexer et al. 2010). However, combined with breeding stochasticity, this process often drives asymmetric directional adaptive introgression, a pattern frequently observed in our retrieved studies (see Takahashi et al. 2017). Our meta‐analysis revealed a strong prevalence of directional introgression, particularly in scenarios where native species introduced adaptive alleles into colonizers, consistent with species expansion events (see Currat et al. 2008; Excoffier et al. 2008). Adaptive alleles introduced into colonizer species from native species that are more abundant and better adapted to local conditions can help the colonizer species establish in the new environment (see Garcia‐Elfring et al. 2017). Our results support this, showing that native‐to‐colonizer gene flow facilitates adaptation to novel environments, aiding new population establishment. Our review also identified cases where mate choice drove adaptive introgression from colonizers to natives, particularly when stochasticity was not the main driver. Colonizer‐to‐native introgression was less common but still significant in our findings, often associated with cases where environmental pressures favored traits introduced by the colonizers. The meta‐analysis results show that balancing selection is closely linked to demographic asymmetries (native‐to‐colonizer or colonizer‐to‐native), likely allowing introgressed alleles to establish in the receptor species' gene pool (Fijarczyk et al. 2018), counteracting genetic drift, genetic swamping, and enabling the persistence of adaptive traits, regardless of abundance imbalances. These findings suggest that demographic asymmetries, alongside ecological factors, play a key role in shaping introgression patterns, especially in dynamic environments where species interactions are simultaneously influenced by abundance and adaptive needs.

#### 4.4.6 Sexual Selection Versus Assortative Mating

The process of mate selection plays a key role in shaping divergence and adaptive introgression patterns. Assortative mating positively impacts divergent evolution by promoting mate choice toward conspecifics, counteracting adaptive introgression. However, our meta‐analysis found adaptive introgression co‐occurring with assortative mating (Lexer et al. 2010; Mavárez et al. 2006; Ng et al. 2017). These findings show that introgression in such cases often involves alleles linked to mate recognition, highlighting the complex role of assortative mating in adaptive introgression. These alleles may mediate the introgression of neighboring genes linked to traits indirectly targeted by adaptive introgression (Mavárez et al. 2006).

Our review found that sexual selection favors mates with higher fitness, regardless of species, potentially influencing the direction and extent of adaptive introgression, a key pattern in our meta‐analysis (Alex Sotola et al. 2019; Salazar et al. 2010). Our findings indicate that sexual selection plays a pivotal role in shaping adaptive introgression, particularly when favoring high‐fitness traits, facilitating gene flow across species boundaries. Sexual promiscuity likely contributes to this process (Baldassarre et al. 2014), spreading novel traits through selective sweeps (see Bay and Ruegg 2017). Under expansion scenarios, sexual selection tends to favor native species, better adapted to local conditions (see Yang et al. 2018). This process can provide an adaptive advantage to the colonizer species by facilitating the incorporation of introgressed adaptive genes from native species (see Zielinski et al. 2013). This pattern aligns with our meta‐analysis, showing that introgressed native alleles often enhance colonizer adaptability to novel environments. However, environmental changes in contact zones may favor the characteristics of colonizer species, opening the path for expansion. From a theoretical perspective, such characteristics can be the target of sexual selection by native species, allowing them to adapt to environmental changes and redirecting adaptive introgression, helping to justify the evidence for adaptive introgression occurring in both directions in the reviewed literature, in species under sexual selection. Our findings support the hypothesis that sexual selection can significantly influence adaptive introgression, particularly in species with intricate mating preferences. The greater prevalence of sexual selection in more complex species underscores the importance of behavioral factors in facilitating gene flow across species.

Other behaviors under selective processes, such as differential parental care and sociality, may also favor asymmetric directional introgression (Sullivan et al. 2016; Trigo et al. 2014). Finally, intra‐sexual interspecific competition, an important node in our networks, can mediate adaptive introgression, promoting the passing of genes from the species with greater competitive capacity (larger size, stronger bite, earlier flowering, etc.) to the other species, thus further contributing to asymmetric directional introgression. Intra‐sexual interspecific competition was thought to occur only under weak or no sexual selection (While et al. 2015). However, our results indicate that intra‐sexual competition often complements sexual selection, with both acting together to shape the balance between adaptive introgression and divergence forces, particularly in competitive mating systems.

#### 4.4.7 Environmental Impacts on Adaptive Introgression

Adaptive introgression may promote adaptation to environmental conditions by increasing genetic variation, which in turn influences evolvability. As shown by Grant and Grant (2016), introgression can drive adaptation by introducing beneficial traits while also being itself an outcome of selective pressures that shape morphological and genetic convergence under natural conditions. Our results further support this dual role of introgression in evolutionary processes. Environmental conditions mediate the iteration between periods of divergence and convergence (Pauquet et al. 2018; Pfennig 2007; Staubach et al. 2012). Based on our review of the literature, both natural environmental factors and anthropogenic impacts influence adaptive introgression through, for example, adaptive responses to antibiotics (see McAshan et al. 1999), pesticides (see Song et al. 2011), deforestation (see Alexander et al. 2017), or climate change (see De La Torre et al. 2014; Quigley et al. 2019). In turn, changes in altitude (see Huerta‐Sánchez et al. 2014), pollinators (see Wang et al. 2013), pathogens exposure (see Jagoda et al. 2018), ultraviolet light (see Ding et al. 2013), and temperature (see Suarez‐Gonzalez et al. 2016) can modulate the strength of ecological post‐breeding barriers (see Reidenbach et al. 2012) and promote variation in hybrid and backcross fitness through adaptive introgression (Kingston et al. 2017). Independently of its extent and historical context, adaptive introgression mediated by environmental conditions helps species to expand their ranges into new ecological regions (Aleksic et al. 2018) and/or can allow them to adapt to new environmental conditions beyond their original niche (De La Torre et al. 2014; Quigley et al. 2019). Differences in local environmental conditions over the evolution of two divergent lineages may help explain spatial discrepancies in the characteristics of adaptive introgression between different contact zones (Stuckas et al. 2009). Finally, allele surfing enables species to spread introgressed alleles throughout the species' range (Sarver et al. 2017), increasing the geographical extent of adaptive introgression beyond the boundaries of contact zones. In our review, we found examples of this phenomenon, for example, in insects that can spread introgressed alleles over 400 km (see Norris et al. 2015) and mammals more than 2000 km from the contact zone in just a few generations (see Matosiuk et al. 2014). These results suggest that environmental pressures act as significant drivers of adaptive introgression. Our findings highlight the role of ecological factors in modulating gene flow, supporting the hypothesis that introgressed alleles can confer adaptive advantages in changing environments.

### 4.5 Limitations

Given the nature of this study, the assumption that the data in our dataset represented adaptively introgressed alleles or traits is based on the interpretation of the authors of papers retrieved from the systematic review, which may carry certain limitations.

First, balancing selection and adaptive introgression can leave similar genomic footprints, such as excess intermediate‐frequency polymorphisms flanking loci under selection. This overlap complicates the differentiation between these processes in many studies. While some original studies relied on sweep patterns or other genomic signals to infer adaptive introgression, others may not have accounted for balancing selection as a confounding factor. As such, the conclusions of our meta‐analysis might, in some cases, include instances of balancing selection rather than adaptive introgression, particularly in genomic regions where both processes co‐occur. Other misinterpretations can occur if researchers overlook alternative explanations for allele sharing between different lineages, such as incomplete lineage sorting (especially in older papers). On the other hand, when interpreting patterns of adaptive introgression, it is essential to account for the role of deleterious mutations, particularly in the context of admixture from large to small populations, as they can confound the identification of regions under selection (Kim et al. 2018). While some methods still ignore deleterious variants, more than 75% of the papers included in our study were published after 2012, a period marked by significant methodological advancements, including genomic and statistical tools, that enhance the accuracy in distinguishing adaptive introgression from other genetic processes. Additionally, our systematic review carefully screened studies for methodological rigor and evidence strength, aiming to minimize the inclusion of studies where alternative mechanisms to introgression could not be reasonably ruled out or where deleterious variants were ostensibly overlooked. The large sample size of studies analyzed further reinforces the robustness and reliability of the observed patterns in adaptive introgression.

Additionally, some misinterpretations may include instances where introgressed alleles are incorrectly assumed to be adaptive without sufficient evidence. These misinterpretations could potentially impact our dataset by introducing bias or inaccuracies in the conclusions drawn regarding the mechanisms and role of adaptive introgression in evolutionary processes. However, while there may be instances where introgression processes are published as adaptive but later determined to be neutral, the high prevalence of studies indicating evidence of exogenous mediation of the introgression process suggests that it primarily occurs in regions of the genome under positive selection. Nonetheless, we emphasize the importance for researchers to critically evaluate the evidence supporting adaptive outcomes of introgression and consider alternative explanations to ensure the robustness of their findings.

### 4.6 Knowledge Gaps and Future Challenges

The advent of high‐throughput sequencing approaches led to the proliferation of empirical examples of allele sharing between different taxa, particularly after 2012, in addition to enabling the identification of the process leading to its establishment (e.g., introgression or incomplete lineage sorting). The fusion of this knowledge about the general characteristics of introgression has allowed us to interpret the patterns and characteristics of the process that leads to adaptation more comprehensively. This has provided an overview of the main hypotheses to be tested regarding the evolutionary role of introgression, which remains a crucial challenge. For example, the introduction of genetic variants through adaptive introgression can be particularly important in the evolutionary process of species facing inbreeding depression, which needs deeper investigation in the current literature.

Our study identified several evolutionary mechanisms related to adaptive introgression that occur concurrently with forces of divergence. This underscores that these processes are not mutually exclusive, even when they act in opposing directions. However, how this balance is established remains unknown in many cases. For example, how might islands of differentiation on sex‐linked chromosomes, during adaptive introgression processes, impact the reproductive performance of the heterogametic sex? This impact extends beyond directly promoting sterility (or even complete unviability) potentially encompassing other endogenous physiological changes in reproductive cells, and/or secondary sexual characteristics (Walsh et al. 2016).

Our study also contributed to identifying the primary mechanisms of adaptive introgression described in the literature, which include a variety of processes co‐occurring with divergence forces. However, the specific consequences of some of these mechanisms are still not fully understood. For example, how do genetic drift and balancing selection influence the occurrence of islands of differentiation outside of sex‐linked chromosomes? Answering this question will help clarify their relevance for adaptive introgression and divergence, which are still not fully understood. Moreover, in cases where colonizing species prefer mating with native species possessing traits better adapted to local environmental conditions, could adaptive introgression confer an advantage to the colonizing species via sexual selection? Our study demonstrated that this pattern is much more common in the literature compared to the opposite. However, there remains a scarcity of examples elucidating the relationship between sexual selection and adaptive introgression from native to colonizer species. Finally, how extensive is the geographic spread of introgressed alleles beyond contact zones? Despite a few interesting examples, little evidence exists of the geographic expansion of introgressed alleles beyond contact zones. Allele surfing was rarely tested and only supported circumstantially. Nonetheless, it is essential to clarify the real spatial extent of adaptive introgression beyond contact zones, as this can provide valuable insights for conservation strategies, species management, and predicting evolutionary responses to environmental changes.

Finally, while our review identifies key trends in the interaction between adaptive introgression and structural organismal characteristics, the role of genomic architecture requires further exploration. Structural variations, such as inversions, fusions, and translocations, can facilitate adaptive introgression by preserving regions under selection; yet they also act as barriers to gene flow by limiting recombination. Similarly, genetic incompatibilities, particularly on sex chromosomes, strongly influence introgression patterns by modulating hybrid fitness. Future studies should prioritize high‐resolution genomic analyses to quantify these effects systematically across taxa and to clarify the balance between facilitating and constraining forces of structural genomic features in adaptive introgression.

Addressing research biases across taxonomic groups, such as the overrepresentation of mammals, angiosperms, and well‐studied genera like *Mus*, *Iris*, *Heliconius*, or *Anopheles*, is essential for a more balanced understanding of adaptive introgression. Broadening the taxonomic scope in future studies may reveal patterns and mechanisms that are currently underexplored.

### 4.7 Applied Implications

Our findings highlight the importance of adaptive introgression as a mechanism that can enhance species' resilience and adaptability, particularly under environmental pressures, which is crucial for both evolutionary biology and conservation strategies. The study's evidence of adaptive introgression functioning across a complexity gradient—from prokaryotes to mammals—underscores its relevance in diverse ecological contexts and biological layers, from genomic to behavioral adaptations. For example, the identified pattern of adaptive introgression on autosomes alongside differentiation in sex‐linked chromosomes suggests a nuanced evolutionary balance between gene flow and species divergence. This knowledge is directly applicable in managing hybridization impacts in conservation biology, particularly for species in fragmented or changing habitats where maintaining adaptive genetic diversity is critical.

The study also points to significant applied potential in predicting evolutionary responses to rapid environmental changes, such as climate change and habitat loss. The observed prevalence of adaptive introgression mediated by balancing selection, especially in ecological contexts like pest resistance or adaptation to temperature extremes, illustrates how introgressed alleles can enable species to rapidly adapt to novel environments. These insights could be leveraged in conservation genetics to support population resilience, offering a framework for anticipating adaptive responses and managing introgressive gene flow in conservation contexts.

## 5 Conclusions

Our meta‐analysis of knowledge trends on adaptive introgression shows that the genomic revolution, post‐2012, helped clarify the characteristics of adaptive introgression and provided evidence for the simultaneous occurrence of counterforces of divergence. We found that the number of published characteristics of adaptive introgression increased from bacteria to mammals across a complexity gradient, progressively occurring in a greater number of levels of biological organization (from genomes to ecology), impacted by the constraints imposed by divergent forces. Testing the tendencies in the published literature, we highlight several co‐existent characteristics of adaptive introgression and some forces of divergence, suggesting they are not mutually exclusive even when they act in opposing directions. This balance should be mediated by environmental conditions regardless of the organisms' structural complexity and the level of biological organization in which these counteracting forces operate. Knowledge gaps persist on the mechanisms behind the balance between convergent and divergent forces and how they drive the capacity of species to adapt to environmental changes. These insights hold important applied implications, particularly for conservation strategies aimed at maintaining adaptive diversity under environmental pressures.

## Conflicts of Interest

The authors declare no conflicts of interest.

## Supporting information


Data S1.

## Data Availability

Data associated with this manuscript should the manuscript be accepted will be archived in the Dryad Digital Repository.

## References

[eva70103-bib-0001] Acosta, M. C. , and A. C. Premoli . 2018. “Understanding the Extensive Hybridization in South American Nothofagus Through Karyotype Analysis.” Botanical Journal of the Linnean Society 188: 74–86.

[eva70103-bib-0002] Aeschbacher, S. , J. P. Selby , J. H. Willis , and G. Coop . 2017. “Population‐Genomic Inference of the Strength and Timing of Selection Against Gene Flow.” Proceedings of the National Academy of Sciences of the United States of America 114: 7061–7066. 28634295 10.1073/pnas.1616755114 PMC5502586

[eva70103-bib-0003] Aleksic, J. M. , S. Skondric , and D. Lakusic . 2018. “Comparative Phylogeography of Capitulate Campanula Species From the Balkans, With Description of a New Species, C‐Daucoides.” Plant Systematics and Evolution 304: 549–575.

[eva70103-bib-0004] Alex Sotola, V. , D. S. Ruppel , T. H. Bonner , C. C. Nice , and N. H. Martin . 2019. “Asymmetric Introgression Between Fishes in the Red River Basin of Texas Is Associated With Variation in Water Quality.” Ecology and Evolution 9: 2083–2095. 30847094 10.1002/ece3.4901 PMC6392354

[eva70103-bib-0005] Alexander, A. M. , Y. C. Su , C. H. Oliveros , K. V. Olson , S. L. Travers , and R. M. Brown . 2017. “Genomic Data Reveals Potential for Hybridization, Introgression, and Incomplete Lineage Sorting to Confound Phylogenetic Relationships in an Adaptive Radiation of Narrow‐Mouth Frogs.” Evolution: International Journal of Organic Evolution 71: 475–488. 27886369 10.1111/evo.13133

[eva70103-bib-0006] Antelope, C. X. , D. Marnetto , F. Casey , and E. Huerta‐Sanchez . 2017. “Leveraging Multiple Populations Across Time Helps Define Accurate Models of Human Evolution: A Reanalysis of the Lactase Persistence Adaptation.” Human Biology 89: 81–97. 29285971 10.13110/humanbiology.89.1.05

[eva70103-bib-0007] Arnold, M. L. 1992. “Natural Hybridization as an Evolutionary Process.” Annual Review of Ecology and Systematics 23: 237–261.

[eva70103-bib-0008] Arnold, M. L. 2004. “Transfer and Origin of Adaptations Through Natural Hybridization: Were Anderson and Stebbins Right?” Plant Cell 16: 562–570. 15004269 10.1105/tpc.HistPersp PMC540259

[eva70103-bib-0009] Arnold, M. L. , E. S. Ballerini , A. N. Brothers , J. A. P. Hamlin , C. D. A. Ishibashi , and M. P. Zuellig . 2012. “The Genomics of Natural Selection and Adaptation: Christmas Past, Present and Future(?).” Plant Ecology and Diversity 5: 451–456.

[eva70103-bib-0148] Baker, N. 2016. “Eugenics and Racial Hygiene: The Connections between the United States and Germany.” Student Theses, Papers and Projects (History) 67. http://digitalcommons.wou.edu/his/67.

[eva70103-bib-0010] Baldassarre, D. T. , T. A. White , J. Karubian , and M. S. Webster . 2014. “Genomic and Morphological Analysis of a Semipermeable Avian Hybrids Zone Suggests Asymmetrical Introgression of a Sexual Signal.” Evolution: International Journal of Organic Evolution 68: 2644–2657. 24889818 10.1111/evo.12457

[eva70103-bib-0011] Bay, R. A. , and K. Ruegg . 2017. “Genomic Islands of Divergence or Opportunities for Introgression?” Proceedings of the Royal Society B: Biological Sciences 284: 20162414. 10.1098/rspb.2016.2414 PMC5360917 28275143

[eva70103-bib-0012] Beresford, J. , M. Elias , L. Pluckrose , et al. 2017. “Widespread Hybridization Within Mound‐Building Wood Ants in Southern Finland Results in Cytonuclear Mismatches and Potential for Sex‐Specific Hybrid Breakdown.” Molecular Ecology 26: 4013–4026. 28503905 10.1111/mec.14183

[eva70103-bib-0150] Biermann, C. , and B. Mansfield . 2014. “Biodiversity, Purity, and Death: Conservation Biology as Biopolitics.” Environment and Planning D: Society and Space 32: 257–273.

[eva70103-bib-0013] Bolnick, D. I. , P. Amarasekare , M. S. Araújo , et al. 2011. “Why Intraspecific Trait Variation Matters in Community Ecology.” Trends in Ecology & Evolution 26: 183–192. 21367482 10.1016/j.tree.2011.01.009 PMC3088364

[eva70103-bib-0014] Bolnick, D. I. , and P. Nosil . 2007. “Natural Selection in Populations Subject to a Migration Load.” Evolution: International Journal of Organic Evolution 61: 2229–2243. 17767592 10.1111/j.1558-5646.2007.00179.x

[eva70103-bib-0015] Bonnet, T. , R. Leblois , F. Rousset , and P. A. Crochet . 2017. “A Reassessment of Explanations for Discordant Introgressions of Mitochondrial and Nuclear Genomes.” Evolution: International Journal of Organic Evolution 71: 2140–2158. 28703292 10.1111/evo.13296

[eva70103-bib-0016] Bradley, R. , J. Greene , E. Russ , L. Dutra , and D. Westen . 2005. “A Multidimensional Meta‐Analysis of Psychotherapy for PTSD.” American Journal of Psychiatry 162: 214–227. 15677582 10.1176/appi.ajp.162.2.214

[eva70103-bib-0017] Bradshaw, H. D. , and D. W. Schemske . 2003. “Allele Substitution at a Flower Colour Locus Produces a Pollinator Shift in Monkeyflowers.” Nature 426, no. 6963: 176–178. 10.1038/nature02106. 14614505

[eva70103-bib-0018] Brennan, A. C. , S. A. Harris , and S. J. Hiscock . 2013. “The Population Genetics of Sporophytic Sel‐Incompatibility in Three Hybridizing Senecio (Asteracea) Species With Contrasting Population Histories.” Evolution: International Journal of Organic Evolution 67: 1347–1367. 23617913 10.1111/evo.12033

[eva70103-bib-0019] Brower, A. V. Z. 2018. “Alternative Facts: A Reconsideration of Putatively Natural Interspecific Hybrid Specimens in the Genus Heliconius (Lepidoptera: Nymphalidae).” Zootaxa 4499: 1–87. 30486085 10.11646/zootaxa.4499.1.1

[eva70103-bib-0020] Brown, A. P. , K. L. McGowan , E. J. Schwarzkopf , et al. 2019. “Local Ancestry Analysis Reveals Genomic Convergence in Extremophile Fishes.” Philosophical Transactions of the Royal Society of London. Series B, Biological Sciences 374: 20180240. 31154969 10.1098/rstb.2018.0240 PMC6560267

[eva70103-bib-0021] Burton, R. S. , and F. S. Barreto . 2012. “A Disproportionate Role for mtDNA in Dobzhansky‐Muller Incompatibilities?” Molecular Ecology 21: 4942–4957. 22994153 10.1111/mec.12006

[eva70103-bib-0022] Cahill, J. A. , I. Stirling , L. Kistler , et al. 2015. “Genomic Evidence of Geographically Widespread Effect of Gene Flow From Polar Bears Into Brown Bears.” Molecular Ecology 24: 1205–1217. 25490862 10.1111/mec.13038 PMC4409089

[eva70103-bib-0023] Carling, M. D. , and R. T. Brumfield . 2008. “Haldane's Rule in Avian System: Using Cline Theory and Divergence Population Genetics to Test for Differential Introgression on Mitochondrial, Autosomal, and Sex‐Linked Loci Across the Passerine Bunting Hybrids Zone.” Evolution: International Journal of Organic Evolution 62: 2600–2615. 18691261 10.1111/j.1558-5646.2008.00477.x

[eva70103-bib-0151] Chan, W. Y. , A. A. Hoffmann , and M. J. H. van Oppen . 2019. “Hybridization as a Conservation Management Tool.” Conservation Letters 12: e12652.

[eva70103-bib-0024] Cruickshank, T. E. , and M. W. Hahn . 2014. “Reanalysis Suggests That Genomic Islands of Speciation Are due to Reduced Diversity, Not Reduced Gene Flow.” Molecular Ecology 23: 3133–3157. 24845075 10.1111/mec.12796

[eva70103-bib-0025] Currat, M. , M. Ruedi , R. J. Petit , and L. Excoffier . 2008. “The Hidden Side of Invasions: Massve Introgression by Local Genes.” Evolution: International Journal of Organic Evolution 62: 1908–1920. 18452573 10.1111/j.1558-5646.2008.00413.x

[eva70103-bib-0026] De Domenico, M. , M. A. Porter , and A. Arenas . 2014. “MuxViz: A Tool for Multilayer Analysis and Visualization of Networks.” Journal of Complex Networks 3: 159–176.

[eva70103-bib-0027] De Domenico, M. , A. Solé‐Ribalta , E. Omodei , S. Gómez , and A. Arenas . 2015. “Ranking in Interconnected Multilayer Networks Reveals Versatile Nodes.” Nature Communications 6: 6868. 10.1038/ncomms7868 25904405

[eva70103-bib-0028] De La Torre, A. R. , T. L. Wang , B. Jaquish , and S. N. Aitken . 2014. “Adaptation and Exogenous Selection in a *Picea glauca* x *Picea engelmannii* Hybrid Zone: Implications for Forest Management Under Climate Change.” New Phytologist 201: 687–699. 24200028 10.1111/nph.12540 PMC4285121

[eva70103-bib-0029] de Lafontaine, G. , J. Prunier , S. Gerardi , and J. Bousquet . 2015. “Tracking the Progression of Speciation: Variable Patterns of Introgression Across the Genome Provide Insights on the Species Delimitation Between Progenitor‐Derivative Spruces (*Picea mariana* x P‐Rubens).” Molecular Ecology 24: 5229–5247. 26346701 10.1111/mec.13377

[eva70103-bib-0030] Derr, J. N. , P. W. Hedrick , N. D. Halbert , et al. 2012. “Phenotypic Effects of Cattle Mitochondrial DNA in American Bison.” Conservation Biology: The Journal of the Society for Conservation Biology 26: 1130–1136. 22862781 10.1111/j.1523-1739.2012.01905.x

[eva70103-bib-0031] Di Candia, M. R. , and E. J. Routman . 2007. “Cytonuclear Discordance Across a Leopard Frog Contact Zone.” Molecular Phylogenetics and Evolution 45: 564–575. 17689987 10.1016/j.ympev.2007.06.014

[eva70103-bib-0032] Ding, Q. , Y. Hu , S. Xu , J. Wang , and L. Jin . 2013. “Neanderthal Introgression at Chromosome 3p21.31 Was Under Positive Natural Selection in East Asians.” Molecular Biology and Evolution 31: 683–695. 24336922 10.1093/molbev/mst260

[eva70103-bib-0033] Dopman, E. B. , L. Pérez , S. M. Bogdanowicz , and R. G. Harrison . 2005. “Consequences of Reproductive Barriers for Genealogical Discordance in the European Corn Borer.” Proceedings of the National Academy of Sciences of the United States of America 102: 14706–14711. 16204000 10.1073/pnas.0502054102 PMC1253542

[eva70103-bib-0034] Dunn, B. , T. Paulish , A. Stanbery , et al. 2013. “Recurrent Rearrangement During Adaptive Evolution in an Interspecific Yeast Hybrid Suggests a Model for Rapid Introgression.” PLoS Genetics 9: 16. 10.1371/journal.pgen.1003366 PMC3605161 23555283

[eva70103-bib-0035] Excoffier, L. , M. Foll , and R. J. Petit . 2008. “Genetic Consequences of Range Expansions.” Annual Review of Ecology, Evolution, and Systematics 40: 481–501.

[eva70103-bib-0036] Falagas, M. E. , E. I. Pitsouni , G. A. Malietzis , and G. Pappas . 2008. “Comparison of PubMed, Scopus, Web of Science, and Google Scholar: Strengths and Weaknesses.” FASEB Journal 22: 338–342. 17884971 10.1096/fj.07-9492LSF

[eva70103-bib-0037] Feder, J. L. , S. P. Egan , and P. Nosil . 2012. “The Genomics of Speciation‐With‐Gene‐Flow.” Trends in Genetics 28, no. 7: 342–350. 22520730 10.1016/j.tig.2012.03.009

[eva70103-bib-0038] Fijarczyk, A. , K. Dudek , M. Niedzicka , and W. Babik . 2018. “Balancing Selection and Introgression of Newt Immune‐Response Genes.” Proceedings of the Royal Society B: Biological Sciences 285: 20180819. 10.1098/rspb.2018.0819 PMC6111169 30111606

[eva70103-bib-0149] Fredrickson, G. M. 2002. “Racism ‐ A Short History.” REV ‐ Revised edn. Princeton University Press.

[eva70103-bib-0039] Gagnaire, P. A. , E. Normandeau , and L. Bernatchez . 2012. “Comparative Genomics Reveals Adaptive Protein Evolution and a Possible Cytonuclear Incompatibility Between European and American Eels.” Molecular Biology and Evolution 29: 2909–2919. 22362081 10.1093/molbev/mss076

[eva70103-bib-0040] Gagnaire, P. A. , S. A. Pavey , E. Normandeau , and L. Bernatchez . 2013. “The Genetic Architecture of the Reproductive Isolation During Speciation‐With‐Gene‐Flow in Lake Whitefish Species Pairs Assessed by RAD Sequencing.” Evolution: International Journal of Organic Evolution 67: 2483–2497. 24033162 10.1111/evo.12075

[eva70103-bib-0041] Garcia‐Elfring, A. , R. D. H. Barrett , M. Combs , T. J. Davies , J. Munshi‐South , and V. Millien . 2017. “Admixture on the Northern Front: Population Genomics of Range Expansion in the White‐Footed Mouse ( *Peromyscus leucopus* ) and Secondary Contact With the Deer Mouse ( *Peromyscus maniculatus* ).” Heredity 119: 447–458. 28902189 10.1038/hdy.2017.57 PMC5677999

[eva70103-bib-0042] Ghenu, A. H. , A. Blanckaert , R. K. Butlin , J. Kulmuni , and C. Bank . 2018. “Conflict Between Heterozygote Advantage and Hybrid Incompatibility in Haplodiploids (And Sex Chromosomes).” Molecular Ecology 27, no. 19: 3935–3949. 10.1111/mec.14482. 29328538

[eva70103-bib-0043] Giannoulis, T. , D. Plageras , C. Stamatis , et al. 2019. “Islands and Hybrid Zones: Combining the Knowledge From “Natural Laboratories” to Explain Phylogeographic Patterns of the European Brown Hare.” BMC Evolutionary Biology 19: 17. 30630408 10.1186/s12862-019-1354-y PMC6329171

[eva70103-bib-0044] Gompert, Z. , and C. A. Buerkle . 2009. “A Powerful Regression‐Based Method for Admixture Mapping of Isolation Across the Genome of Hybrids.” Molecular Ecology 18: 1207–1224. 19243513 10.1111/j.1365-294X.2009.04098.x

[eva70103-bib-0045] Gompert, Z. , L. K. Lucas , C. C. Nice , and C. A. Buerkle . 2013. “Genome Divergence and the Genetic Architecture of Barriers to Gene Flow Between Lycaeides Idas and *L. melissa* .” Evolution: International Journal of Organic Evolution 67: 2498–2514. 24033163 10.1111/evo.12021

[eva70103-bib-0046] Grant, P. R. , and B. R. Grant . 2016. “Introgressive Hybridization and Natural Selection in Darwin's Finches.” Biological Journal of the Linnean Society 117: 812–822.

[eva70103-bib-0047] Haldane, J. B. S. 1922. “Sex Ratio and Unisexual Sterility in Hybrid Animals.” Journal of Genetics 12: 101–109.

[eva70103-bib-0049] Harrison, R. G. , and E. L. Larson . 2014. “Hybridization, Introgression, and the Nature of Species Boundaries.” Journal of Heredity 105: 795–809. 25149255 10.1093/jhered/esu033

[eva70103-bib-0050] Hawkins, M. T. R. , J. A. Leonard , K. M. Helgen , M. M. McDonough , L. L. Rockwood , and J. E. Maldonado . 2016. “Evolutionary History of Endemic Sulawesi Squirrels Constructed From UCEs and Mitogenomes Sequenced From Museum Specimens.” BMC Evolutionary Biology 16: 16. 27075887 10.1186/s12862-016-0650-z PMC4831120

[eva70103-bib-0051] Hayden, E. C. 2014. “Technology: The $1,000 Genome.” Nature 507: 294–295. 24646979 10.1038/507294a

[eva70103-bib-0052] Hedrick, P. W. 2013. “Adaptive Introgression in Animals: Examples and Comparison to New Mutation and Standing Variation as Sources of Adaptive Variation.” Molecular Ecology 22: 4606–4618. 23906376 10.1111/mec.12415

[eva70103-bib-0152] Henkel, J. , R. Saif , V. Jagannathan , et al. 2019. “Selection Signatures in Goats Reveal Copy Number Variants Underlying Breed‐Defining Coat Color Phenotypes.” PLoS Genetics 15: e1008536. 31841508 10.1371/journal.pgen.1008536 PMC6936872

[eva70103-bib-0053] Herbers, J. M. 2010. “Evolution: Fundamentals.” In Encyclopedia of Animal Behavior, edited by M. D. Breed and J. Moore , 670–678. Academic Press.

[eva70103-bib-0054] Herrig, D. K. , A. J. Modrick , E. Brud , and A. Llopart . 2014. “Introgression in the Drosophila Subobscura— *D. madeirensis* Sister Species: Evidence of Gene Flow in Nuclear Genes Despite Mitochondrial Differentiation.” Evolution: International Journal of Organic Evolution 68: 705–719. 24152112 10.1111/evo.12295 PMC4255303

[eva70103-bib-0055] Hoffmann, A. A. , and L. H. Rieseberg . 2008. “Revisiting the Impact of Inversions in Evolution: From Population Genetics to Species Formation.” Annual Review of Ecology, Evolution, and Systematics 39, no. 1: 21–42. 10.1146/annurev.ecolsys.39.110707.173532 PMC2858385 20419035

[eva70103-bib-0057] Huerta‐Sánchez, E. , X. Jin , B. Asan , et al. 2014. “Altitude Adaptation in Tibetans Caused by Introgression of Denisovan‐Like DNA.” Nature 512: 194–197. 25043035 10.1038/nature13408 PMC4134395

[eva70103-bib-0058] Hulsey, C. D. , B. P. Keck , H. Alamillo , and B. C. O'Meara . 2013. “Mitochondrial Genome Primers for Lake Malawi Cichlids.” Molecular Ecology Resources 13: 347–353. 23347464 10.1111/1755-0998.12066

[eva70103-bib-0059] Hume, J. B. , H. Recknagel , C. W. Bean , C. E. Adams , and B. K. Mable . 2018. “RADseq and Mate Choice Assays Reveal Unidirectional Gene Flow Among Three Lamprey Ecotypes Despite Weak Assortative Mating: Insights Into the Formation and Stability of Multiple Ecotypes in Sympatry.” Molecular Ecology 27: 4572–4590. 30252984 10.1111/mec.14881

[eva70103-bib-0060] Jagoda, E. , D. J. Lawson , J. D. Wall , et al. 2018. “Disentangling Immediate Adaptive Introgression From Selection on Standing Introgressed Variation in Humans.” Molecular Biology and Evolution 35, no. 3: 623–630. 10.1093/molbev/msx314. 29220488 PMC5850494

[eva70103-bib-0061] James, K. E. , H. Schneider , S. W. Ansell , et al. 2008. “Diversity Arrays Technology (DArT) for Pan‐Genomic Evolutionary Studies of Non‐Model Organisms.” PLoS One 3, no. 2: e1682. 18301759 10.1371/journal.pone.0001682 PMC2244804

[eva70103-bib-0062] Jones, M. R. , L. S. Mills , P. C. Alves , et al. 2018. “Adaptive Introgression Underlies Polymorphic Seasonal Camouflage in Snowshoe Hares.” Science (New York, N.Y.) 360: 1355–1358. 29930138 10.1126/science.aar5273

[eva70103-bib-0063] Kim, B. Y. , C. D. Huber , and K. E. Lohmueller . 2018. “Deleterious Variation Shapes the Genomic Landscape of Introgression.” PLoS Genetics 14, no. 10: e1007741. 30346959 10.1371/journal.pgen.1007741 PMC6233928

[eva70103-bib-0064] King, R. C. , W. D. Stansfield , and P. K. Mulligan . 2006. A Dictionary of Genetics. Oxford University Press.

[eva70103-bib-0065] Kingston, S. E. , T. L. Parchman , Z. Gompert , C. A. Buerkle , and M. J. Braun . 2017. “Heterogeneity and Concordance in Locus‐Specific Differentiation and Introgression Between Species of Towhees.” Journal of Evolutionary Biology 30: 474–485. 28009485 10.1111/jeb.13033

[eva70103-bib-0066] Kuhlwilm, M. , S. Han , V. C. Sousa , L. Excoffier , and T. Marques‐Bonet . 2019. “Ancient Admixture From an Extinct Ape Lineage Into Bonobos.” Nature Ecology & Evolution 3: 957–965. 31036897 10.1038/s41559-019-0881-7

[eva70103-bib-0067] Kulikova, I. V. , Y. N. Zhuravlev , and K. G. McCracken . 2004. “Asymmetric Hybridization and Sex‐Biased Gene Flow Between Eastern Spot‐Billed Ducks ( *Anas zonorhyncha* ) and Mallards ( *A. platyrhynchos* ) in the Russian Far East.” Auk 121: 930–949.

[eva70103-bib-0068] Kulmuni, J. , and P. Pamilo . 2014. “Introgression in Hybrid Ants Is Favored in Females but Selected Against in Males.” Proceedings of the National Academy of Sciences of the United States of America 111: 12805–12810. 25136088 10.1073/pnas.1323045111 PMC4156693

[eva70103-bib-0069] Latch, E. K. , L. A. Harveson , J. S. King , M. D. Hobson , and O. E. Rhodes . 2006. “Assessing Hybridization in Wildlife Populations Using Molecular Markers: A Case Study in Wild Turkeys.” Journal of Wildlife Management 70: 485–492.

[eva70103-bib-0070] Lexer, C. , B. Heinze , R. Alia , and L. H. Rieseberg . 2004. “Hybrid Zones as a Tool for Identifying Adaptive Genetic Variation in Outbreeding Forest Trees: Lessons From Wild Annual Sunflowers (*Helianthus* spp.).” Forest Ecology and Management 197: 49–64. 18677413 10.1016/j.foreco.2004.05.004 PMC2493040

[eva70103-bib-0071] Lexer, C. , J. A. Joseph , M. van Loo , et al. 2010. “Genomic Admixture Analysis in European Populus spp. Reveals Unexpected Patterns of Reproductive Isolation and Mating.” Genetics 186: 699–712. 20679517 10.1534/genetics.110.118828 PMC2954470

[eva70103-bib-0072] Liu, K. J. , E. Steinberg , A. Yozzo , Y. Song , M. H. Kohn , and L. Nakhleh . 2015. “Interspecific Introgressive Origin of Genomic Diversity in the House Mouse.” Proceedings of the National Academy of Sciences of the United States of America 112: 196–201. 25512534 10.1073/pnas.1406298111 PMC4291675

[eva70103-bib-0073] Llopart, A. , D. Herrig , E. Brud , and Z. Stecklein . 2014. “Sequential Adaptive Introgression of the Mitochondrial Genome in Drosophila Yakuba and Drosophila Santomea.” Molecular Ecology 23: 1124–1136. 24460929 10.1111/mec.12678 PMC4260671

[eva70103-bib-0074] Maguilla, E. , M. Escudero , A. L. Hipp , and M. Luceno . 2017. “Allopatric Speciation Despite Historical Gene Flow: Divergence and Hybridization in Carex Furva and C.Lucennoiberica (Cyperaceae) Inferred From Plastid and Nuclear RAD‐Seq Data.” Molecular Ecology 26: 5646–5662. 28742230 10.1111/mec.14253

[eva70103-bib-0075] Mallet, J. 2005. “Hybridization as an Invasion of the Genome.” Trends in Ecology & Evolution 20, no. 5: 229–237. 16701374 10.1016/j.tree.2005.02.010

[eva70103-bib-0076] Maroco, J. 2010. Análise Estatística Com Utilização do SPSS. 3ª edição ed. Edições Silabo.

[eva70103-bib-0077] Martinsen, G. D. , T. G. Whitham , R. J. Turek , and P. Keim . 2001. “Hybrid Populations Selectively Filter Gene Introgression Between Species.” Evolution: International Journal of Organic Evolution 55: 1325–1335. 11525457 10.1111/j.0014-3820.2001.tb00655.x

[eva70103-bib-0078] Matosiuk, M. , I. N. Sheremetyeva , I. S. Sheremetyev , A. P. Saveljev , and A. Borkowska . 2014. “Evolutionary Neutrality of mtDNA Introgression: Evidence From Complete Mitogenome Analysis in Roe Deer.” Journal of Evolutionary Biology 27: 2483–2494. 25262616 10.1111/jeb.12491

[eva70103-bib-0079] Mavárez, J. , C. A. Salazar , E. Bermingham , C. Salcedo , C. D. Jiggins , and M. Linares . 2006. “Speciation by Hybridization in Heliconius Butterflies.” Nature 441: 868–871. 16778888 10.1038/nature04738

[eva70103-bib-0080] Mayr, E. 1942. Systematics and the Origin of Species From the Viewpoint of a Zoologist. Columbia University Press.

[eva70103-bib-0081] Mazzocchi, F. 2008. “Complexity in Biology. Exceeding the Limits of Reductionism and Determinism Using Complexity Theory.” EMBO Reports 9: 10–14. 18174892 10.1038/sj.embor.7401147 PMC2246621

[eva70103-bib-0082] McAshan, S. K. , K. L. Vergin , S. J. Giovannoni , and D. S. Thaler . 1999. “Interspecies Recombination Between Enterococci: Genetic and Phenotypic Diversity of Vancomycin‐Resistant Transconjugants.” Microbial Drug Resistance (Larchmont, NY) 5: 101–112. 10.1089/mdr.1999.5.101 10432271

[eva70103-bib-0083] McDonald, D. B. , R. P. Clay , R. T. Brumfield , and M. J. Braun . 2001. “Sexual Selection on Plumage and Behavior in an Avian Hybrid Zone: Experimental Tests of Male‐Male Interactions.” Evolution: International Journal of Organic Evolution 55: 1443–1451. 11525466 10.1111/j.0014-3820.2001.tb00664.x

[eva70103-bib-0084] Minder, A. M. , and A. Widmer . 2008. “A Population Genomic Analysis of Species Boundaries: Neutral Processes, Adaptive Divergence and Introgression Between Two Hybridizing Plant Species.” Molecular Ecology 17: 1552–1563. 18321255 10.1111/j.1365-294X.2008.03709.x

[eva70103-bib-0085] Moher, D. , A. Liberati , J. Tetzlaff , D. G. Altman , and P. G. The . 2009. “Preferred Reporting Items for Systematic Reviews and Meta‐Analyses: The PRISMA Statement.” PLoS Medicine 6: e1000097. 19621072 10.1371/journal.pmed.1000097 PMC2707599

[eva70103-bib-0086] Morales, H. E. , A. Pavlova , N. Amos , et al. 2018. “Concordant Divergence of Mitogenomes and a Mitonuclear Gene Cluster in Bird Lineages Inhabiting Different Climates.” Nature Ecology & Evolution 2: 1258–1267. 29988164 10.1038/s41559-018-0606-3

[eva70103-bib-0087] Morales, H. E. , P. Sunnucks , L. Joseph , and A. Pavlova . 2017. “Perpendicular Axes of Differentiation Generated by Mitochondrial Introgression.” Molecular Ecology 26: 3241–3255. 28329425 10.1111/mec.14114

[eva70103-bib-0088] Muirhead, C. A. , and D. C. Presgraves . 2016. “Hybrid Incompatibilities, Local Adaptation, and the Genomic Distribution of Natural Introgression Between Species.” American Naturalist 187: 249–261. 10.1086/684583 26807751

[eva70103-bib-0089] Nachman, M. W. , and B. A. Payseur . 2012. “Recombination Rate Variation and Speciation: Theoretical Predictions and Empirical Results From Rabbits and Mice.” Philosophical Transactions of the Royal Society B 367, no. 1587: 409–421. 10.1098/rstb.2011.0249 PMC3233716 22201170

[eva70103-bib-0090] Nadachowska‐Brzyska, K. , P. Zieliński , J. Radwan , and W. Babik . 2012. “Interspecific Hybridization Increases MHC Class II Diversity in Two Sister Species of Newts.” Molecular Ecology 21: 887–906. 22066802 10.1111/j.1365-294X.2011.05347.x

[eva70103-bib-0091] Nader, J. L. , T. C. Mathers , B. J. Ward , et al. 2019. “Evolutionary Genomics of Anthroponosis in Cryptosporidium.” Nature Microbiology 4: 826–836. 10.1038/s41564-019-0377-x 30833731

[eva70103-bib-0092] Navarro, A. , and N. H. Barton . 2003. “Chromosomal Speciation and Molecular Divergence—Accelerated Evolution in Rearranged Chromosomes.” Science 300, no. 5617: 321–324. 12690198 10.1126/science.1080600

[eva70103-bib-0093] Neri, J. , T. Wendt , and C. Palma‐Silva . 2017. “Natural Hybridization and Genetic and Morphological Variation Between Two Epiphytic Bromeliads.” AoB Plants 10, no. 1: plx061. 29308124 10.1093/aobpla/plx061 PMC5751037

[eva70103-bib-0094] Ng, N. S. R. , P. R. Wilton , D. M. Prawiradilaga , et al. 2017. “The Effects of Pleistocene Climate Change on Biotic Differentiation in a Montane Songbird Glade From Wallacea.” Molecular Phylogenetics and Evolution 114: 353–366. 28501612 10.1016/j.ympev.2017.05.007

[eva70103-bib-0095] Nichols, P. , M. J. Genner , C. van Oosterhout , et al. 2015. “Secondary Contact Seeds Phenotypic Novelty in Cichlid Fishes.” Proceedings of the Royal Society B: Biological Sciences 282: 20142272. 10.1098/rspb.2014.2272 PMC4262179 25392475

[eva70103-bib-0096] Norris, L. C. , B. J. Main , Y. Lee , et al. 2015. “Adaptive Introgression in an African Malaria Mosquito Coincident With the Increased Usage of Insecticide‐Treated Bed Nets.” Proceedings of the National Academy of Sciences of the United States of America 112: 815–820. 25561525 10.1073/pnas.1418892112 PMC4311837

[eva70103-bib-0097] Nosil, P. , T. L. Parchman , J. L. Feder , and Z. Gompert . 2012. “Do Highly Divergent Loci Reside in Genomic Regions Affecting Reproductive Isolation? A Test Using Next‐Generation Sequence Data in Timema Stick Insects.” BMC Evolutionary Biology 12: 164. 22938057 10.1186/1471-2148-12-164 PMC3502483

[eva70103-bib-0098] O'Dea, R. E. , M. Lagisz , M. D. Jennions , et al. 2021. “Preferred Reporting Items for Systematic Reviews and Meta‐Analyses in Ecology and Evolutionary Biology: A PRISMA Extension.” Biological Reviews 96: 1695–1722. 33960637 10.1111/brv.12721 PMC8518748

[eva70103-bib-0099] Oliver, P. , J. A. Castro , A. Picornell , et al. 2002. “Linkage Disequilibria Between mtDNA Haplotypes and Chromosomal Arrangements in a Natural Population of Drosophila Subobscura.” Heredity 89: 133–138. 12136416 10.1038/sj.hdy.6800116

[eva70103-bib-0100] Page, M. J. , J. E. McKenzie , P. M. Bossuyt , et al. 2021. “The PRISMA 2020 Statement: An Updated Guideline for Reporting Systematic Reviews.” BMJ 372: n71. 10.1136/bmj.n71. 33782057 PMC8005924

[eva70103-bib-0101] Palmer, K. L. , V. N. Kos , and M. S. Gilmore . 2010. “Horizontal Gene Transfer and the Genomics of Enterococcal Antibiotic Resistance.” Current Opinion in Microbiology 13: 632–639. 20837397 10.1016/j.mib.2010.08.004 PMC2955785

[eva70103-bib-0102] Pardo‐Diaz, C. , C. Salazar , S. W. Baxter , et al. 2012. “Adaptive Introgression Across Species Boundaries in Heliconius Butterflies.” PLoS Genetics 8, no. 6: 13. 10.1371/journal.pgen.1002752. PMC3380824 22737081

[eva70103-bib-0103] Parrett, J. M. , and R. J. Knell . 2018. “The Effect of Sexual Selection on Adaptation and Extinction Under Increasing Temperatures.” Proceedings of the Biological Sciences 285, no. 1877: 20180303. 10.1098/rspb.2018.0303. 29669902 PMC5936732

[eva70103-bib-0104] Pauquet, G. , W. Salzburger , and B. Egger . 2018. “The Puzzling Phylogeography of the Haplochromine Cichlid Fish *Astatotilapia burtoni* .” Ecology and Evolution 8: 5637–5648. 29938080 10.1002/ece3.4092 PMC6010872

[eva70103-bib-0105] Payseur, B. A. , J. G. Krenz , and M. W. Nachman . 2004. “Differential Patterns of Introgression Across the X Chromosome in a Hybrid Zone Between Two Species of House Mice.” Evolution: International Journal of Organic Evolution 58: 2064–2078. 15521462 10.1111/j.0014-3820.2004.tb00490.x

[eva70103-bib-0106] Pease, J. B. , D. C. Haak , M. W. Hahn , and L. C. Moyle . 2016. “Phylogenomics Reveals Three Sources of Adaptive Variation During a Rapid Radiation.” PLoS Biology 14: 24. 10.1371/journal.pbio.1002379 PMC4752443 26871574

[eva70103-bib-0107] Pfeifer, B. , and D. D. Kapan . 2019. “Estimates of Introgression as a Function of Pairwise Distances.” BMC Bioinformatics 20: 207. 31014244 10.1186/s12859-019-2747-z PMC6480520

[eva70103-bib-0108] Pfennig, K. S. 2007. “Facultative Mate Choice Drives Adaptive Hybridization.” Science (New York, N.Y.) 318: 965–967. 17991861 10.1126/science.1146035

[eva70103-bib-0109] Pfennig, K. S. 2021. “Biased Hybridization and Its Impact on Adaptive Introgression.” Trends in Ecology & Evolution 36: 488–497. 33752896 10.1016/j.tree.2021.02.010

[eva70103-bib-0110] Powell, J. R. , V. Petrarca , A. della Torre , A. Caccone , and M. Coluzzi . 1999. “Population Structure, Speciation, and Introgression in the *Anopheles Gambiae* Complex.” Parassitologia 41: 101–113. 10697841

[eva70103-bib-0111] Pujolar, J. M. , M. W. Jacobsen , T. D. Als , et al. 2014. “Genome‐Wide Single‐Generation Signatures of Local Selection in the Panmictic European Eel.” Molecular Ecology 23: 2514–2528. 24750353 10.1111/mec.12753

[eva70103-bib-0112] Quigley, K. M. , L. K. Bay , and M. J. H. van Oppen . 2019. “The Active Spread of Adaptive Variation for Reef Resilience.” Ecology and Evolution 9: 11122–11135. 31641460 10.1002/ece3.5616 PMC6802068

[eva70103-bib-0113] Räsänen, K. , and A. P. Hendry . 2008. “Disentangling Interactions Between Adaptive Divergence and Gene Flow When Ecology Drives Diversification.” Ecology Letters 11: 624–636. 18384363 10.1111/j.1461-0248.2008.01176.x

[eva70103-bib-0114] Ray, N. , and L. Excoffier . 2009. “Inferring Past Demography Using Spatially Explicit Population Genetic Models.” Human Biology 81: 141–157. 19943741 10.3378/027.081.0303

[eva70103-bib-0115] Reidenbach, K. R. , D. E. Neafsey , C. Costantini , et al. 2012. “Patterns of Genomic Differentiation Between Ecologically Differentiated M and S Forms of *Anopheles Gambiae* in West and Central Africa.” Genome Biology and Evolution 4: 1202–1212. 23132896 10.1093/gbe/evs095 PMC3542583

[eva70103-bib-0116] Rieseberg, L. H. , O. Raymond , D. M. Rosenthal , et al. 2003. “Major Ecological Transitions in Wild Sunflowers Facilitated by Hybridization.” Science (New York, N.Y.) 301: 1211–1216. 12907807 10.1126/science.1086949

[eva70103-bib-0117] Runemark, A. , C. N. Trier , F. Eroukhmanoff , et al. 2018. “Variation and Constraints in Hybrid Genome Formation.” Nature Ecology & Evolution 2: 549–556. 29335572 10.1038/s41559-017-0437-7

[eva70103-bib-0118] Sackton, T. B. , H. Montenegro , D. L. Hartl , and B. Lemos . 2011. “Interspecific Y Chromosome Introgressions Disrupt Testis‐Specific Gene Expression and Male Reproductive Phenotypes in *Drosophila* .” Proceedings of the National Academy of Sciences 108: 17046–17051. 10.1073/pnas.1114690108 PMC3193250 21969588

[eva70103-bib-0119] Salazar, C. , S. W. Baxter , C. Pardo‐Diaz , et al. 2010. “Genetic Evidence for Hybrid Trait Speciation in Heliconius Butterflies.” PLoS Genetics 6: e1000930. 20442862 10.1371/journal.pgen.1000930 PMC2861694

[eva70103-bib-0121] Sarver, B. A. J. , J. R. Demboski , J. M. Good , N. Forshee , S. S. Hunter , and J. Sullivan . 2017. “Comparative Phylogenomic Assessment of Mitochondrial Introgression Among Several Species of Chipmunks (Tamias).” Genome Biology and Evolution 9: 7–19. 28172670 10.1093/gbe/evw254 PMC5381575

[eva70103-bib-0124] Seixas, F. A. , P. Boursot , and J. Melo‐Ferreira . 2018. “The Genomic Impact of Historical Hybridization With Massive Mitochondrial DNA Introgression.” Genome Biology 19: 91. 30056805 10.1186/s13059-018-1471-8 PMC6065068

[eva70103-bib-0125] Shamseer, L. , D. Moher , M. Clarke , et al. 2015. “Preferred Reporting Items for Systematic Review and Meta‐Analysis Protocols (PRISMA‐P) 2015: Elaboration and Explanation.” BMJ: British Medical Journal 349: g7647. 10.1136/bmj.g7647 25555855

[eva70103-bib-0126] Song, Y. , S. Endepols , N. Klemann , et al. 2011. “Adaptive Introgression of Anticoagulant Rodent Poison Resistance by Hybridization Between Old World Mice.” Current Biology 21: 1296–1301. 21782438 10.1016/j.cub.2011.06.043 PMC3152605

[eva70103-bib-0127] Staubach, F. , A. Lorenc , P. W. Messer , K. Tang , D. A. Petrov , and D. Tautz . 2012. “Genome Patterns of Selection and Introgression of Haplotypes in Natural Populations of the House Mouse ( *Mus musculus* ).” PLoS Genetics 8: e1002891. 22956910 10.1371/journal.pgen.1002891 PMC3431316

[eva70103-bib-0128] Storchová, R. , S. Gregorová , D. Buckiová , V. Kyselová , P. Divina , and J. Forejt . 2004. “Genetic Analysis of X‐Linked Hybrid Sterility in the House Mouse.” Mammalian Genome 15: 515–524. 15366371 10.1007/s00335-004-2386-0

[eva70103-bib-0129] Stuckas, H. , K. Stoof , H. Quesada , and R. Tiedemann . 2009. “Evolutionary Implications of Discordant Clines Across the Baltic Mytilus Hybrid Zone (Mytilus Edulis and *Mytilus trossulus* ).” Heredity 103: 146–156. 19384341 10.1038/hdy.2009.37

[eva70103-bib-0130] Suarez‐Gonzalez, A. , C. A. Hefer , C. Christe , et al. 2016. “Genomic and Functional Approaches Reveal a Case of Adaptive Introgression From *Populus balsamifera* (Balsam Poplar) in *P. trichocarpa* (Black Cottonwood).” Molecular Ecology 25: 2427–2442. 26825293 10.1111/mec.13539

[eva70103-bib-0131] Sullivan, A. R. , S. A. Owusu , J. A. Weber , A. L. Hipp , and O. Gailing . 2016. “Hybridization and Divergence in Multi‐Species Oak (Quercus) Communities.” Botanical Journal of the Linnean Society 181: 99–114.

[eva70103-bib-0132] Sun, Y. , P. Corcoran , A. Menkis , C. A. Whittle , S. G. E. Andersson , and H. Johannesson . 2012. “Large‐Scale Introgression Shapes the Evolution of the Mating‐Type Chromosomes of the Filamentous Ascomycete Neurospora Tetrasperma.” PLoS Genetics 8: 19. 10.1371/journal.pgen.1002820 PMC3406010 22844246

[eva70103-bib-0133] Takahashi, H. , A. Toyoda , T. Yamazaki , S. Narita , T. Mashiko , and Y. Yamazaki . 2017. “Asymmetric Hybridization and Introgression Between Sibling Species of the Pufferfish Takifugu That Have Undergone Explosive Speciation.” Marine Biology 164: 11.

[eva70103-bib-0134] Taylor, S. A. , and E. L. Larson . 2019. “Insights From Genomes Into the Evolutionary Importance and Prevalence of Hybridization in Nature.” Nature Ecology & Evolution 3: 170–177. 30697003 10.1038/s41559-018-0777-y

[eva70103-bib-0135] Taylor, S. J. , M. Arnold , and N. H. Martin . 2009. “The Genetic Architecture of Reproductive Isolation in Louisiana Irises: Hybrids Fitness in Nature.” Evolution: International Journal of Organic Evolution 63: 2581–2594. 19549289 10.1111/j.1558-5646.2009.00742.x

[eva70103-bib-0136] Todesco, M. , M. A. Pascual , G. L. Owens , et al. 2016. “Hybridization and Extinction.” Evolutionary Applications 9: 892–908. 27468307 10.1111/eva.12367 PMC4947151

[eva70103-bib-0137] Trigo, T. C. , F. P. Tirelli , T. R. O. de Freitas , and E. Eizirik . 2014. “Comparative Assessment of Genetic and Morphological Variation at an Extensive Hybrid Zone Between Two Wild Cats in Southern Brazil.” PLoS One 9: 15. 10.1371/journal.pone.0108469 PMC4177223 25250657

[eva70103-bib-0139] Vaillant, J. J. , D. G. Bock , G. D. Haffner , and M. E. Cristescu . 2013. “Speciation Patterns and Processes in the Zooplankton of the Ancient Lakes of Sulawesi Island, Indonesia.” Ecology and Evolution 3: 3083–3094. 24101996 10.1002/ece3.697 PMC3790553

[eva70103-bib-0140] Van Valen, L. 1976. “Ecological Species, Multispecies, and Oaks.” Taxon 25: 233–239.

[eva70103-bib-0141] Wade, M. J. , and C. J. Goodnight . 2006. “Cyto‐Nuclear Epistasis: Two‐Locus Random Genetic Drift in Hermaphroditic and Dioecious Species.” Evolution: International Journal of Organic Evolution 60: 643–659. 16739448

[eva70103-bib-0142] Wadsworth, C. B. , X. Li , and E. B. Dopman . 2015. “A Recombination Suppressor Contributes to Ecological Speciation in OSTRINIA Moths.” Heredity 114: 593–600. 25626887 10.1038/hdy.2014.128 PMC4434251

[eva70103-bib-0143] Walsh, J. , W. G. Shriver , B. J. Olsen , and A. I. Kovach . 2016. “Differential Introgression and the Maintenance of Species Boundaries in an Advanced Generation Avian Hybrid Zone.” BMC Evolutionary Biology 16: 18. 27000833 10.1186/s12862-016-0635-y PMC4802838

[eva70103-bib-0144] Wang, H. Y. , C. H. Hsieh , C. G. Huang , et al. 2013. “Genetic and Physiological Data Suggest Demographic and Adaptive Responses in Complex Interactions Between Populations of Figs ( *Ficus pumila* ) and Their Pollinating Wasps (Wiebesia Pumilae).” Molecular Ecology 22: 3814–3832. 23841862 10.1111/mec.12336

[eva70103-bib-0145] While, G. M. , S. Michaelides , R. J. P. Heathcote , et al. 2015. “Sexual Selection Drives Asymmetric Introgression in Wall Lizards.” Ecology Letters 18: 1366–1375. 26468006 10.1111/ele.12531

[eva70103-bib-0146] Yang, W. Z. , G. M. While , H. Laakkonen , et al. 2018. “Genomic Evidence for Asymmetric Introgression by Sexual Selection in the Common Wall Lizard.” Molecular Ecology 27: 4213–4224. 30192998 10.1111/mec.14861

[eva70103-bib-0147] Zielinski, P. , K. Nadachowska‐Brzyska , B. Wielstra , et al. 2013. “No Evidence for Nuclear Introgression Despite Complete mtDNA Replacement in the Carpathian Newt ( *Lissotriton montandoni* ).” Molecular Ecology 22: 1884–1903. 23379646 10.1111/mec.12225

