# Supplementary material for: Adaptive Introgression as an Evolutionary Force: A Meta‐Analysis of Knowledge Trends
Source: Evol Appl. 2025 Jun 20;18(6):e70103. doi: 10.1111/eva.70103 (PMC12181397; doi:10.1111/eva.70103)
Supplement: Supplementary file 1 — Data S1. [file EVA-18-e70103-s001.docx]

Supplementary Material for

**Adaptive Introgression as an Evolutionary force: a Meta-analysis of Knowledge Trends**

**This file includes:**

Supplementary Text

WebFig. S1

Figs. S1 and S2

Tables S1, S2, S3

**Identification of studies via other methods**

**Identification of studies via databases and registers**

Records identified from:

Citation searching (n = 47)

Records removed *before screening*:

Duplicate records removed (n = 279)

Records identified from:

Databases (n = 820)

**Identification**

Records screened

(n = 541)

Records excluded

(n = 55)

Reports not retrieved

(n = 20)

Reports sought for retrieval

(n = 47)

Reports sought for retrieval

(n = 486)

Reports not retrieved

(n = 77)

**Screening**

Reports assessed for eligibility

(n = 27)

Reports excluded:

Reason 1 (n = 2)

Reason 2 (n = 9)

Reports assessed for eligibility

(n = 409)

Reports excluded:

Reason 1 (n = 20)

Reason 2 (n = 47)

.

Studies included in review

(n = 358)

**Included**

**WebFigure 1.** PRISMA 2020 flow diagram. *Adapted from:*  Page MJ, McKenzie JE, Bossuyt PM, Boutron I, Hoffmann TC, Mulrow CD, et al. The PRISMA 2020 statement: an updated guideline for reporting systematic reviews. BMJ 2021;372:n71. doi: 10.1136/bmj.n71. For more information, visit: <http://www.prisma-statement.org/>

**Search details**

We used the following search string for on-line databases:

“Topic=(*hybrid** AND *adapt** AND *introgress** AND *genom** NOT *invas** NOT *domest** NOT *exotic*) OR Topic=(*adapt** AND *introgress** AND *genom** NOT *invas** NOT *domest** NOT *exotic*)”

We defined some keywords as strict retention criteria: Adapt* (all terms related to adaptation, adaptive, etc.), Introgress* (all terms related to introgression, introgressive, etc.) and Genom* (all terms related to genome, genomics, etc.). We also used as non-strict retention criterion for the term hybrid* (all terms related to hybrids, hybridization, etc.) that could be present together with the previous ones or not. We excluded all breeding contacts between taxa under direct human interference (breeding manipulations between species that do not naturally interbreed, genetic manipulation, invasive species scenarios, etc.). We used the following keywords as strict exclusion criteria: Invas* (all terms related to invasion, invasive species, etc.), Exotic and Domest* (all terms related to domestic species, domestication, etc.).

We defined the expression “*Adaptive Introgression*” as a keyword used as strict retention criteria searching for relevance grey literature through Google and Google scholar. We extracted 43 different variables grouped into seven categories: 1) Paper bibliometric information; 2) Type of publication; 3) Taxa under study; 4) Epoch and location of the introgressions case studies; 5) Methods used; 6) Sub-mechanisms of introgression and 7) Outcomes.

**Table S1**. Full list of information and the total number of papers that includes each category extracted from each reviewed paper.

| Categories of information | Variables extracted | Classes used | Number of papers |
| --- | --- | --- | --- |
| Bibliographic information | - Authors - Year - Title - Journal - Research area - Type of publication - Location of first author’s institution |  | 358  358  358  358  358  358  358 |
| Authors’ initial perception about introgression | - Polarity - Why positive? - Why negative? - Why neutral? - Prevalence - Semantics | Positive,  Negative,  Both,  Neutral.  Very rare [-3],  Rare [-2],  Infrequent [-1],  Unknown [0],  Few cases [1],  Some cases [2],  Common [3],  Many cases [4],  Ubiquitous [5].  Hybrid* terms used,  Hybrid* terms not used. | 351  250  78  129  135  358 |
| Taxa under study | - Main taxonomic group - Families - Species pairs | Animal,  Plant,  Fungi,  Protists,  Bacteria. | 355  355  355 |
| Time and space of introgressions’ case studies | - Epoch - Continent - Approximated location | Ancient,  Recent,  Both. | 355  355  355 |
| Methods used | - Type of experiment - Type of introgression markers - Whole-genome sequencing - Reference genome | In-situ,  Ex-situ,  Both.  Quantitative Trait Locus,  Microsatellites,  Mitochondrial DNA,  Ribosomal DNA,  Single-Nucleotide,  Polymorphisms,  AFLP,  Karyotypes.  performed,  Not performed.  Available,  Not available. | 351  347  347  165 |
| Sub-mechanisms | - Cyto-nuclear location of islands of speciation - Nuclear location of islands of speciation - Cyto-nuclear location of introgression - Nuclear location of introgression - Sex-biased introgression - Haldane’s rule - Maternal vs paternal way - Sexual selection impacts - Assortative mating preferences - Intra-sex interspecific competition - Balancing selection - Demographic impacts - Symmetry - Direction - Donor species - Receptor species - Native-to-colonizer - Endogenous impacts - Exogenous impacts - Climate-mediated introgression - Allele surfing | Cytoplasm (mitochondria, ribosome, chloroplast), Nucleus;  Both.  Sex-linked chromosomes, Autosomes,  Both.  Cytoplasm (mitochondria, ribosome, chloroplast), Nucleus;  Both.  Sex-linked chromosomes, Autosomes,  Both.  Yes,  No.  Yes,  No.  Maternal,  Paternal.  Yes,  No.  Yes,  No.  Yes,  No.  Yes (Multiallelic, transespecific polymorphisms, etc.),  No (Genetic drift).  Yes,  No.  Symmetric,  Asymmetric.  Unidirectional,  Bidirectional.  Yes,  No.  Yes (Baculum format, flowering time, sexual characters, etc.),  No.  Yes (temperature, topography, pesticides, etc.),  No.  Yes,  No.  Yes,  No. | 87  83  182  91  96  34  91  51  12  22  40  67  276  259  259  259  81  28  64  53  32 |
| Outcomes | - Phenotypic traits shared - Physiologic system - Polarity - Adaptive outcomes | Positive,  Negative,  Neutral. | 230  230  230  230 |

**Table S2.** Quantification of complexity levels across taxonomic groups based on the sum of Binary Classifications for three key evolutionary characteristics: Organizational Unit (Prokaryotic vs. Eukaryotic), Presence of Sex Chromosomes, and Primary Mating Strategy (Random Mating vs. Sexual Selection).

| Taxonomic group | **Cell type** | Binary classification | **Sex-linked chromosomes** | Binary classification | **Mating strategy** | Binary classification | **TOTAL complexity** | Figure 1* correspondence |
| --- | --- | --- | --- | --- | --- | --- | --- | --- |
| Bacilli | prokaryotic | 0 | absent | 0 | random | 0 | 0 | A |
| Conoidasia | eukaryotic | 1 | absent | 0 | random | 0 | 1 | B |
| Ascomycetes | eukaryotic | 1 | absent | 0 | random | 0 | 1 | B |
| Yeasts | eukaryotic | 1 | absent | 0 | random | 0 | 1 | B |
| Basidiomycetes | eukaryotic | 1 | absent | 0 | random | 0 | 1 | B |
| Euascomycetes | eukaryotic | 1 | absent | 0 | random | 0 | 1 | B |
| Cnidaria | eukaryotic | 1 | present | 1 | random | 0 | 2 | C |
| Mussels | eukaryotic | 1 | present | 1 | random | 0 | 2 | C |
| Crustacea | eukaryotic | 1 | present | 1 | random | 0 | 2 | C |
| Gymnosperms | eukaryotic | 1 | present | 1 | random | 0 | 2 | C |
| Fishes | eukaryotic | 1 | present | 1 | Sexual selection | 1 | 3 | D |
| Insects | eukaryotic | 1 | present | 1 | Sexual selection | 1 | 3 | D |
| Amphibians | eukaryotic | 1 | present | 1 | Sexual selection | 1 | 3 | D |
| Reptiles | eukaryotic | 1 | present | 1 | Sexual selection | 1 | 3 | D |
| Birds | eukaryotic | 1 | present | 1 | Sexual selection | 1 | 3 | D |
| Angiosperms | eukaryotic | 1 | present | 1 | Sexual selection | 1 | 3 | D |
| Mammals | eukaryotic | 1 | present | 1 | Sexual selection | 1 | 3 | D |

*main text


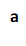


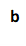


**Figure S1.** Temporal changes in the number of published papers on adaptive introgression by methodology (a) and scientific field (b). The solid lines represent the correlation between the number of papers and time and the dashed line shows the tipping point, when the trend line started to show an exponential increase – representing the beginning of the genomic revolution in adaptive introgression studies.

**Table S3.** Statistical values of multiple comparisons of taxonomic groups (A – Prokaryotes, B - Singled-cell eukaryotes, C -Eukaryotes sex-linked chromosomes and random mate and D - Eukaryotes with sexual selection) on the percentage of connections across the five levels of biological organization ("Genomics/cytology", "Physiology", "Demography", "Behaviour" and "Ecology") according to the post-hoc HSD Tukey test. Green cells represent significant differences (p < 0.05).

|  |  | A | B | C | D |
| --- | --- | --- | --- | --- | --- |
|  |  | Genomics/Cytology | | | |
| A |  |  | 0.345 | 0.001 | 0.003 |
| B | Physiology | 0.868 |  | 0.006 | 0.012 |
| C |  | 0.342 | 0.585 |  | 0.791 |
| D |  | 0.085 | 0.106 | 0.783 |  |
|  |  | A | B | C | D |
|  |  | Behaviour | | | |
| A |  |  | 1.000 | 1.000 | 0.012 |
| B | Demography | 0.962 |  | 1.000 | 0.019 |
| C |  | 0.292 | 0.319 |  | 0.029 |
| D |  | 0.423 | 0.482 | 0.947 |  |
|  |  | A | B | C | D |
|  |  | Ecology | | | |
| A |  |  | 0.853 | 0.134 | 0.685 |
| B |  |  |  | 0.223 | 0.980 |
| C |  |  |  |  | 0.307 |
| D |  |  |  |  |  |


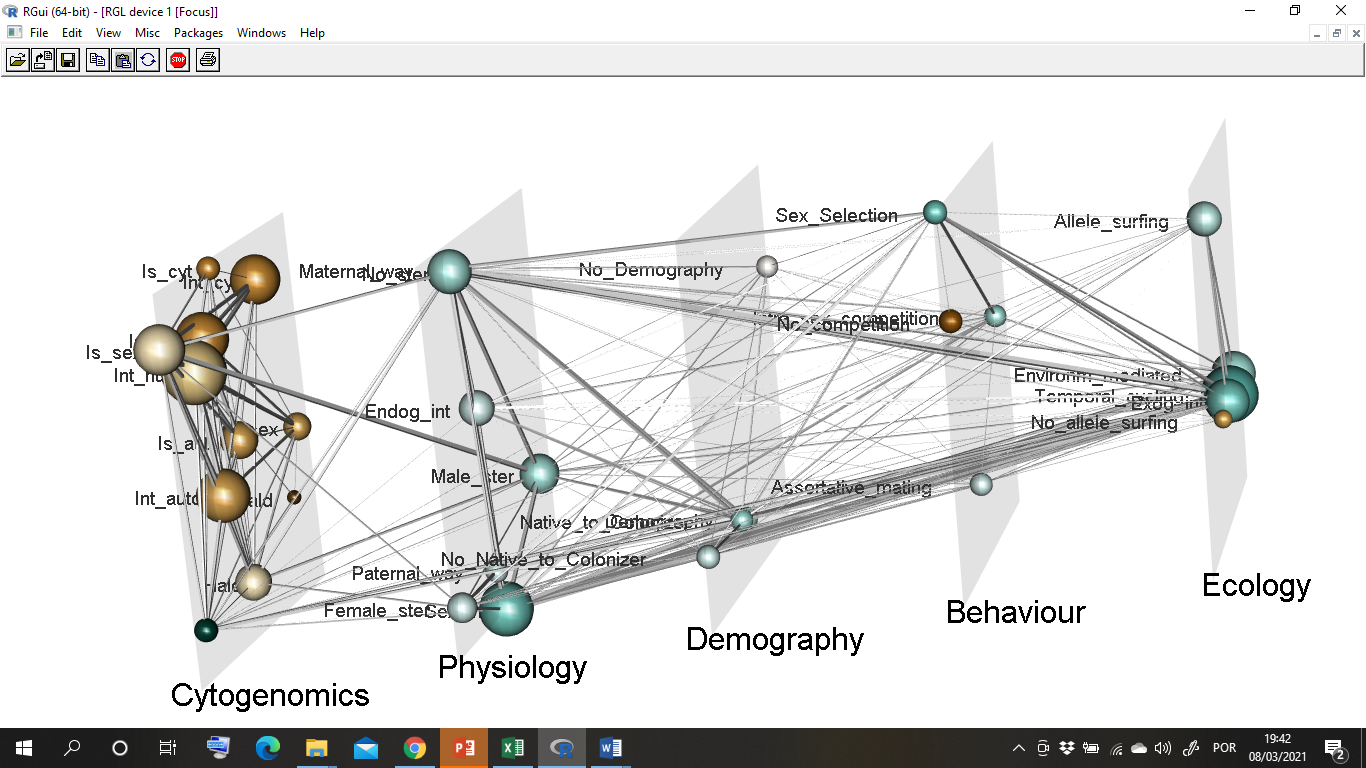


Genomics/cytology


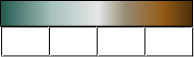


0 0.35 0.5 0.75 1

Node degree

Physiology

Demography

Behaviour

Ecology

**Figure S2**. Multilayer 3D graphs of networks of adaptive introgression’s characteristics at different levels of biological organization.
